# Mulheres Médicas: Burnout durante a Pandemia de COVID-19 no Brasil

**DOI:** 10.36660/abc.20210938

**Published:** 2022-07-07

**Authors:** Gláucia Maria Moraes de Oliveira, Viviana Guzzo Lemke, Maria Sanali Moura de Oliveira Paiva, Giordana Zeferino Mariano, Elizabeth Regina Giunco Alexandre Silva, Sheyla Cristina Tonheiro Ferro da Silva, Magaly Arrais dos Santos, Imara Correia de Queiroz Barbosa, Carla Janice Baister Lantieri, Elizabeth da Rosa Duarte, Maria Cristina Oliveira Izar, Karin Jaeger Anzolch, Milena Alonso Egea Gerez, Mayara Viana de Oliveira Ramos, Maria Antonieta Albanez Albuquerque de Medeiro Lopes, Emilia Matos do Nascimento, Nanette Kass Wenger

**Affiliations:** 1 Universidade Federal do Rio de Janeiro Rio de Janeiro RJ Brasil Universidade Federal do Rio de Janeiro, Rio de Janeiro, RJ – Brasil; 2 Cardiocare Curitiba PR Brasil Cardiocare, Curitiba, PR – Brasil; 3 Hospital Universitário Onofre Lopes Natal RN Brasil Hospital Universitário Onofre Lopes, Natal, RN – Brasil; 4 Hospital São João Batista Criciúma SC Brasil Hospital São João Batista, Criciúma, SC – Brasil; 5 Hospital do Coração São Paulo SP Brasil Hospital do Coração, São Paulo, SP – Brasil; 6 CEMISE Aracaju SE Brasil CEMISE, Aracaju, SE – Brasil; 7 Universidade Federal de Campina Grande Campina Grande PB Brasil Universidade Federal de Campina Grande, Campina Grande, PB – Brasil; 8 Centro Universitário Faculdade de Medicina do ABC Santo André SP Brasil Centro Universitário Faculdade de Medicina do ABC, Santo André, SP – Brasil; 9 Hospital Nossa Senhora da Conceição Porto Alegre RS Brasil Hospital Nossa Senhora da Conceição, Porto Alegre, RS – Brasil; 10 Universidade Federal de São Paulo São Paulo SP Brasil Universidade Federal de São Paulo, São Paulo, SP – Brasil; 11 Hospital Moinhos de Vento Porto Alegre RS Brasil Hospital Moinhos de Vento, Porto Alegre, RS – Brasil; 12 Instituto de Moléstias Cardiovasculares São José do Rio Preto SP Brasil Instituto de Moléstias Cardiovasculares, São José do Rio Preto, SP – Brasil; 13 Universidade Federal do Maranhão São Luis MA Brasil Universidade Federal do Maranhão, São Luis, MA – Brasil; 14 Real Hospital Português Recife PE Brasil Real Hospital Português, Recife, PE – Brasil; 15 Universidade do Estado do Rio de Janeiro Rio de Janeiro RJ Brasil Universidade do Estado do Rio de Janeiro, Rio de Janeiro, RJ – Brasil; 16 Emory University School of Medicine Atlanta Georgia EUA Emory University School of Medicine Atlanta, Georgia – EUA

**Keywords:** Mulheres Médicas, Burnout, Pandemia de COVID-19, Brasil

## Abstract

**Fundamento:**

A COVID-19 adicionou um fardo enorme sobre os médicos ao redor do mundo, especialmente as mulheres médicas, que são afetadas pelo aumento da carga de trabalho e pela perda da qualidade de vida.

**Objetivo:**

Avaliar os efeitos da pandemia de COVID-19 na qualidade de vida, *burnout* e espiritualidade de médicas brasileiras que atendem pacientes com COVID-19 direta ou indiretamente.

**Método:**

Estudo prospectivo, observacional realizado de 28 de julho a 27 de setembro de 2020, no Brasil, com mulheres médicas de 47 especialidades, a mais frequente sendo a cardiologia (22,8%), sem restrição de idade. Elas responderam voluntariamente um questionário online com questões sobre características demográficas e socioeconômicas, qualidade de vida (WHOQOL-brief) e espiritualidade (WHOQOL-SRPB) e enunciados do Oldenburg Burnout Inventory. A análise estatística utilizou o software R, regressão beta, árvores de classificação e matriz de correlação policórica, com nível de significância de 5%.

**Resultados:**

Das 769 respondentes, 61,6% relataram sinais de *burnout.* Cerca de 64% relataram perda salarial de até 50% durante a pandemia. Algumas relataram falta de energia para as tarefas diárias, sentimentos negativos frequentes, insatisfação com a capacidade para o trabalho, e que cuidar de outras pessoas não agregava sentido às suas vidas. Os sentimentos negativos correlacionaram-se negativamente com a satisfação com a vida sexual, a satisfação com as relações pessoais e a energia para as tarefas diárias. A incapacidade de permanecer otimista em tempos de incerteza correlacionou-se positivamente com a sensação de insegurança no dia a dia e com o não reconhecimento de que cuidar de outras pessoas trouxesse sentido à vida.

**Conclusão:**

O presente estudo mostrou uma alta frequência de *burnout* entre as médicas brasileiras que responderam ao questionário durante a pandemia de COVID-19. Apesar disso, apresentavam uma qualidade de vida relativamente boa e acreditavam que a espiritualidade trazia-lhes conforto e segurança nos momentos difíceis.

## Introdução

Os médicos na linha de frente contra a doença de coronavírus 2019 (COVID-19) enfrentaram níveis de estresse altos, sem precedentes. Apesar disso, pouca atenção foi dada à vulnerabilidade vivenciada por esses profissionais, principalmente do sexo feminino. Uma revisão sistemática realizada nos bancos de dados Medline e Embase mostrou um aumento nos desafios relacionados à alta carga de trabalho e à perda de qualidade de vida durante a pandemia de COVID-19, que estão associados à exaustão física e mental.^1^ A prevalência de *burnout* variou de 23% a 76%, e no gênero feminino alta carga de trabalho e preocupações relacionadas à família foram preditores de *burnout* .^1^ Os autores recomendaram que estudos sobre *burnout* em médicos levassem em consideração as diferenças de gênero.

Um estudo, realizando uma pesquisa transversal para avaliar 2.707 profissionais de saúde (PS) de 60 países, relatou que 51% deles apresentavam *burnout* , que estava associado ao trabalho com impacto nas atividades domésticas, exposição a pacientes com COVID-19, treinamento inadequado e tomada de decisões que priorizavam a vida. O *burnout* foi mais frequente em países de alta renda.^2^ Outro estudo relatou um aumento do *burnout* em mulheres médicas em comparação com médicos homens, e os autores levantaram a hipótese de que estressores específicos incluíam falta de opções de creche para crianças e desequilíbrio entre trabalho e vida pessoal. As mulheres médicas com maior carga de trabalho e aquelas sem companheiro apresentaram níveis mais altos de *burnout* .^3^ Vale ressaltar que as mulheres atualmente constituem uma grande proporção da força global de trabalho em saúde e gastam 15 horas a mais por semana em trabalho doméstico não remunerado.^4 , 5^

As dimensões de *burnout* foram significativamente associadas a um risco aumentado para doenças, independentemente de fatores sociodemográficos e sintomas depressivos. Um estudo com 5.671 participantes [predominantemente médicos, idade média de 44,1 anos (variação, 18 a 70 anos), 62,4% mulheres] usou um aplicativo digital de saúde móvel para uma pesquisa online de *burnout* profissional medido com o Maslach Burnout Inventory-General Survey. Por meio de análise de rede e regressão logística, o estudo mostrou a associação de alta exaustão emocional com hipertensão arterial e outras doenças crônicas após ajuste para idade, sexo, escolaridade e sintomas depressivos.^6^

Outra revisão sistemática com 12 estudos avaliando o *burnout* em PS que trabalham ou não nas enfermarias de COVID-19 da linha de frente mostrou resultados controversos.^7^ Dois dos estudos relataram níveis mais altos de fadiga emocional em mulheres em comparação com homens e que o sexo feminino foi um fator de risco para *burnout* entre profissionais de terapia intensiva.^8 , 9^ No entanto, outro estudo não encontrou associação com o gênero.^10^ A heterogeneidade dos estudos quanto à coleta de dados e questionários utilizados pode ter contribuído para isso, o que reforça a necessidade de mais estudos.^7^

O Brasil ocupou o segundo lugar em número de casos e óbitos por COVID-19 desde o começo da pandemia. No entanto, até onde sabemos, nenhum estudo avaliou o *burnout* de mulheres médicas brasileiras durante a pandemia. Assim, este estudo teve como objetivo avaliar os efeitos da pandemia na qualidade de vida, no desenvolvimento do *burnout* e na espiritualidade de mulheres médicas que atendem pacientes com COVID-19 direta ou indiretamente.

## Métodos

Trata-se de um estudo transversal, observacional, realizado de 28 de julho a 27 de setembro de 2020 no Brasil, com mulheres médicas de diferentes especialidades, que prestavam assistência direta ou indireta a pacientes com COVID-19. Não houve restrição de idade. As médicas responderam voluntariamente a um questionário online com 68 questões, assim constituindo uma amostra de conveniência.

O questionário foi composto por: 20 questões sobre características demográficas e socioeconômicas; 26 questões da versão em português brasileiro do WHOQOL-brief;^11^ 9 questões baseadas no instrumento de teste de campo de Qualidade de Vida da Organização Mundial da Saúde – módulo Espiritualidade, Religiosidade e Crenças Pessoais;^12 , 13^ e os 13 enunciados da versão em português brasileiro do Oldenburg Burnout Inventory (OLBI).^14 - 16^ (Material suplementar 1).

Por meio da identificação do usuário, as participantes que respondessem ao questionário várias vezes poderiam ser identificadas. Todas as participantes forneceram consentimento informado para o uso de seus dados anônimos.

O presente estudo foi aprovado pelo Comitê de Ética em Pesquisa (HUOL-CAAE: 34673520.7.0000.5292).

De acordo com a metodologia proposta por Schuster et al. e Demerouti et al., os 13 enunciados sobre *burnout* do OLBI foram transformados em variáveis das duas dimensões de ‘despersonificação’ (7 variáveis) e ‘exaustão emocional’ (6 variáveis).^15 , 17^ Foi invertida a pontuação das questões cujas respostas eram ‘concordo’ ou ‘discordo’ de modo que quanto maior a pontuação de cada variável, maior o nível de *burnout* . A cada dimensão foi atribuída uma pontuação correspondente à sua pontuação média.

### Análise estatística

Foi realizada a análise estatística por meio de regressão beta^18^ que modela taxas e proporções de desfechos. As duas dimensões de *burnout* foram consideradas como desfechos e as 55 questões restantes, como variáveis independentes.

No OLBI, a pontuação de cada desfecho é limitada ao intervalo de 1 a 4. Desta maneira, foi implementado um modelo de regressão beta, onde cada desfecho foi recalculado por meio de interpolação linear, de modo que valores de 0 a 1 pudessem ser obtidos. Foram implementados três modelos para cada desfecho. O primeiro modelo foi composto por 55 variáveis. O segundo e terceiro modelos utilizaram as variáveis independentes que apresentaram significância de 10% no modelo anterior.

Após os modelos de regressão beta, foram implementadas árvores de regressão utilizando as variáveis independentes do modelo final e seus respectivos desfechos. As árvores de classificação e regressão (CART, sigla em inglês) constituem um método não paramétrico utilizado para obter uma associação entre a variável dependente e um conjunto de covariáveis. Árvores de decisão são usadas para identificar a interação entre covariáveis. As folhas da árvore fornecem uma representação gráfica do desfecho para cada grupo de indivíduos. Foram usados os pacotes betareg^18^ e partykit em R para implementar os modelos de regressão beta da árvore de regressão.^19 , 20^

Outra visualização gráfica foi utilizada com base em uma correlação policórica^21^ , uma medida de associação entre variáveis categóricas ordinais. Uma matriz de correlação policórica foi representada como uma rede onde os nós eram as variáveis, e os pesos nas arestas representavam o coeficiente de correlação policórica. A espessura das arestas e a transparência foram dadas pela magnitude do coeficiente de correlação entre os nós. As cores vermelho e verde corresponderam a correlações negativas e positivas, respectivamente. O pacote ‘qgraph’ em R foi utilizado para visualização da rede.^20 - 22^

Para os testes estatísticos, adotamos o nível de significância de 5%.

## Resultados

Das 769 respondentes, 474 (61,6%) relataram sinais de *burnout* . O critério de classificação das respondentes foi dado pelos pontos de corte obtidos das árvores de classificação: exaustão emocional (< 2,668 e ≥ 2,668) e despersonificação (< 2,143 e ≥ 2,143) (Material Suplementar 2).

Com base nas respostas às questões, as características da amostra foram as seguintes: menos de 50 anos, 50,2%; cor da pele branca, 81,9%; casadas, 87,8%; e com 1 a 3 filhos, 67,5%. A distribuição das 47 especialidades médicas foi a seguinte: Cardiologia, 22,8%; Pediatria, 15%; Medicina Interna, 6%; Obstetrícia e Ginecologia, 5,6%; Anestesiologia, 3,8%; Medicina de Família e Comunidade, 2,9%; e Medicina Intensiva, 2,5%. Todas as cinco regiões geográficas brasileiras estavam representadas, sendo mais frequentes as regiões Sudeste (34,3%), Sul (31,7%) e Nordeste (28,3%).

A maioria das respondentes trabalhava em cidades com mais de 500 mil habitantes (74,1%), não ocupava cargo de liderança (66,2%), tinha estabilidade no trabalho (74,5%) e trabalhava em dois ou três locais diferentes (59,7%). Dedicavam de 6 a 20 horas semanais a afazeres domésticos (54,8%) e até 5 horas com atividades de lazer (59,0%). Cerca de 64% das respondentes ganhavam de US$ 1.000 a US$ 4.000 e 57,6% relataram perda salarial de até 50% durante a pandemia, 61% relataram boas condições de trabalho e disponibilidade de equipamentos de proteção individual adequados (61,5%).

A maioria das respondentes relatou ter uma boa qualidade de vida (71,7%) e estar satisfeita com sua saúde (55%), enquanto 64,8% relataram não aproveitar verdadeiramente a vida. Quase 80% relataram acreditar que suas vidas tinham um propósito e 90,4% reconheceram que cuidar de outras pessoas trouxe significado para suas vidas. Consideraram satisfatórios os seguintes aspectos de suas vidas: sono, 62,9%; capacidade de realizar tarefas diárias, 54,7%; capacidade para o trabalho, 64,4%; relações pessoais, 57,7%; apoio de amigos, 61%; condições do lar, 84%; e acesso à saúde, 81,4%. Apenas 36,6% consideraram a sua vida sexual satisfatória e cerca de 94% tiveram, pelo menos ocasionalmente, sentimentos negativos. Apenas 37% relataram sentir energia suficiente para as tarefas diárias e 48,6% aceitaram a sua aparência física.

As respondentes acreditavam que a espiritualidade trazia-lhes conforto e segurança (73,2%) e encontravam força espiritual em tempos difíceis (70,6%), com boa conexão de corpo, mente e espírito (67,8%), embora apenas 53,4% relatassem paz interior e 50,7% relatassem ser otimista. Além disso, 72,7% das respondentes relataram encontrar força na fé e 44,3% encontraram apoio em comunidades religiosas ou espirituais.

As Tabelas 1 e 2 mostram o modelo de regressão beta para os desfechos de ‘exaustão emocional’ e ‘despersonificação’, respectivamente.

**Tabela 1:** Modelo de regressão beta para a dimensão de ‘exaustão emocional’, uma das dimensões de *burnout* (Oldenburg Burnout Inventory)

Variáveis preditivas	Estimativa (IC 95%)	p
Local de trabalho (subúrbio ou arredores de cidade grande)	0,328 (0,139; 0,516)	0,001 ***
Local de trabalho (cidade média)	0,358 (0,105; 0,611)	0,006 **
Local de trabalho (cidade pequena)	-0,265 (-0,571; 0,04)	0,089 .
Alocação de tempo para afazeres domésticos (6-10 horas/semana)	-0,05 (-0,184; 0,084)	0,469
Alocação de tempo para afazeres domésticos (11-20 horas/semana)	-0,139 (-0,259; -0,019)	0,023 *
Alocação de tempo para afazeres domésticos (> 20 horas/semana)	-0,122 (-0,226; -0,018)	0,022 *
Faixa salarial (US$ 500-1000)	0,205 (-0,314; 0,724)	0,439
Faixa salarial (US$ 1000-2000)	-0,374 (-0,816; 0,068)	0,097 .
Faixa salarial (US$ 2000-4000)	0,252 (-0,047; 0,552)	0,099 .
Faixa salarial (> US$ 4000)	-0,233 (-0,412; -0,054)	0,011 *
Ambiente de trabalho (ruim)	-0,479 (-0,806; -0,153)	0,004 **
Ambiente de trabalho (regular)	0,193 (-0,084; 0,471)	0,172
Ambiente de trabalho (bom)	-0,049 (-0,251; 0,152)	0,630
Ambiente de trabalho (excelente)	-0,005 (-0,136; 0,125)	0,937
Qualidade de vida	-0,186 (-0,261; -0,111)	< 0,001 ***
Dor física	0,06 (-0,004; 0,124)	0,064 .
Necessidade de tratamento	0,067 (0,01; 0,124)	0,020 *
Energia	-0,395 (-0,479; -0,311)	< 0,001 ***
Alocação de tempo para lazer	-0,09 (-0,162; -0,019)	0,013 *
Deslocamento para trabalho	0,102 (0,038; 0,167)	0,002 **
Capacidade para o trabalho	-0,183 (-0,258; -0,108)	< 0,001 ***
Satisfação com as relações pessoais	-0,109 (-0,176; -0,042)	0,001 ***
Satisfação com a vida sexual	-0,058 (-0,108; -0,008)	0,023 *
Satisfação com o lar	0,07 (-0,001; 0,142)	0,055 .
Satisfação com o transporte	-0,156 (-0,233; -0,078)	< 0,001 ***
Sentimentos negativos	0,246 (0,181; 0,311)	< 0,001 ***

IC 95%: intervalo de confiança de 95%. Significado dos códigos: 0 ‘***’ 0,001 ‘**’ 0,01 ‘*’ 0,05 ‘.’ 0,1 ‘ ‘ 1

**Tabela 2:** Modelo de regressão beta para a dimensão de ‘despersonificação’, outra dimensão de burnout (Oldenburg Burnout Inventory)

Variáveis preditivas	Estimativa (IC 95%)	p
Estado civil (casada ou com companheiro)	0,231 (0,089; 0,373)	0,001 ***
Estado civil (separada ou divorciada)	0,187 (0,008; 0,366)	0,041 *
Estado civil (viúva)	0,307 (-0,06; 0,674)	0,101
Local de trabalho (subúrbio ou arredores de cidade grande)	0,364 (0,184; 0,544)	< 0,001 ***
Local de trabalho (cidade média)	0,239 (-0,008; 0,485)	0,058 .
Local de trabalho (cidade pequena)	-0,123 (-0,423; 0,177)	0,422
Carga de trabalho (21-36 horas)	-0,045 (-0,194; 0,105)	0,560
Carga de trabalho (37-48 horas)	0,038 (-0,089; 0,166)	0,557
Carga de trabalho (49-60 horas)	-0,051 (-0,161; 0,059)	0,365
Carga de trabalho (> 60 horas)	0,084 (-0,008; 0,177)	0,074 .
Alocação de tempo para afazeres domésticos (6-10 horas/semana)	-0,119 (-0,251; 0,013)	0,078 .
Alocação de tempo para afazeres domésticos (11-20 horas/semana)	-0,164 (-0,281; -0,047)	0,006 **
Alocação de tempo para afazeres domésticos (> 20 horas/semana)	-0,238 (-0,338; -0,139)	< 0,001 ***
Alocação de tempo para lazer (6-10 horas/semana)	0,167 (-0,032; 0,366)	0,101
Alocação de tempo para lazer (11-20 horas/semana)	0,16 (-0,011; 0,33)	0,067 .
Alocação de tempo para lazer (> 20 horas/semana)	0,057 (-0,08; 0,194)	0,417
Salário durante a pandemia (redução de 20%)	-0,141 (-0,282; 0)	0,050 *
Salário durante a pandemia (redução de 21%-50%)	0,016 (-0,119; 0,15)	0,820
Salário durante a pandemia (redução de ≥ 50%)	0,134 (0,006; 0,262)	0,040 *
Salário durante a pandemia (aumento)	0,114 (0,005; 0,223)	0,041 *
Ambiente de trabalho (ruim)	-0,454 (-0,756; -0,153)	0,003 **
Ambiente de trabalho (regular)	0,01 (-0,242; 0,262)	0,938
Ambiente de trabalho (bom)	-0,1 (-0,283; 0,083)	0,283
Ambiente de trabalho (excelente)	0,059 (-0,061; 0,178)	0,336
Dor física	0,082 (0,027; 0,137)	0,004 **
Concentração	-0,144 (-0,226; -0,062)	0,001 ***
Segurança de vida	-0,142 (-0,225; -0,059)	0,001 ***
Ambiente saudável	-0,12 (-0,193; -0,047)	0,001 ***
Energia	-0,201 (-0,283; -0,12)	< 0,001 ***
Aceitação da aparência física	-0,065 (-0,125; -0,005)	0,034 *
Satisfação com a capacidade de realizar tarefas diárias	0,109 (0,031; 0,187)	0,006 **
Capacidade para o trabalho	-0,249 (-0,332; -0,167)	< 0,001 ***
Satisfação com a vida sexual	-0,08 (-0,128; -0,032)	0,001 ***
Cuidar de outras pessoas agrega sentido à vida	-0,236 (-0,311; -0,161)	< 0,001 ***
Otimismo	-0,12 (-0,188; -0,052)	0,001 ***

IC 95%: intervalo de confiança de 95%. Significado dos códigos: 0 ‘***’ 0,001 ‘**’ 0,01 ‘*’ 0,05 ‘.’ 0,1 ‘ ‘ 1

Para o desfecho de ‘exaustão emocional’ ( Tabela 1 ), foram significativos os seguintes: local de trabalho; alocação de tempo para afazeres domésticos; faixa salarial; ambiente de trabalho desfavorável; má qualidade de vida; falta de energia para as tarefas diárias; falta de alocação de tempo para lazer; e insatisfação com o deslocamento diário do trabalho. Relataram insatisfação significativa com sua: capacidade para o trabalho; transporte; habilidades de relacionamento; e vida sexual. Além disso, relataram vivenciar sentimentos negativos com frequência.

Para o desfecho de ‘despersonificação ( Tabela 2 ), foram significativos os seguintes: estado civil; local de trabalho; alocação de tempo para afazeres domésticos; redução/aumento de renda durante a pandemia; ambiente de trabalho ruim; incapacidade de concentração; sentimento diário de insegurança; ambiente físico insalubre; falta de energia para as tarefas diárias; não aceitação da aparência física; grande insatisfação com a capacidade para as tarefas diárias, com a vida sexual e com a capacidade para o trabalho; incapacidade de permanecer otimista em tempos de incerteza; ausência de sentido para a própria vida ao cuidar de outras pessoas; e a consideração de que a dor física as impedia de fazer o que precisava ser feito.

**Figura 1:**
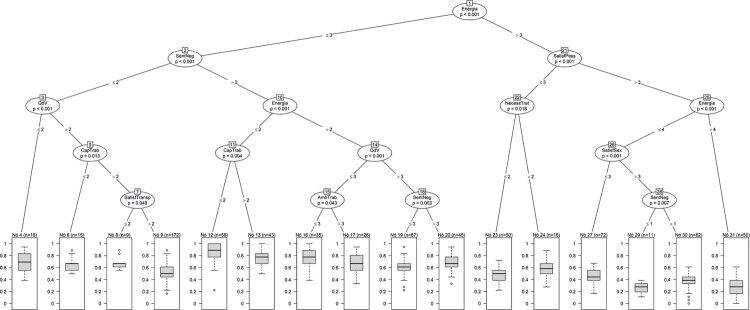
Árvore de classificação correspondendo ao desfecho de ‘exaustão emocional’, uma das dimensões de burnout (Oldenburg Burnout Inventory). AmbTrab: ambiente de trabalho; CapTrab: capacidade para o trabalho; NecessTrat: necessidade de tratamento médico; QdV: qualidade de vida; SentNeg: sentimentos negativos; SatisfPess: satisfação com as relações pessoais; SatisfSex: satisfação com a vida sexual; SatisfTransp: satisfação com o transporte.

Na árvore de classificação correspondente ao desfecho de ‘exaustão emocional’ ( Figura 1 ), as respondentes representadas nas folhas 12 (n = 58, 7,5%), 13 (n = 43, 5,6%) e 16 (n = 35, 4,5%) apresentaram os maiores escores de *burnout* , com média e mediana iguais ou superiores a 3,4 (correspondendo a 0,8 na escala de 0 a 1). As respondentes representadas nas folhas 12 e 13 relataram sentir pouca ou nenhuma energia para as tarefas diárias e sentimentos negativos muito frequentes. Esses dois grupos diferiram quanto à capacidade para o trabalho, e as da folha 12 relataram grande insatisfação com isso. As respondentes da folha 16 relataram sentir pouca energia para as tarefas diárias, nenhum ou muito poucos sentimentos negativos e não ter boa qualidade de vida.

**Figura 2:**
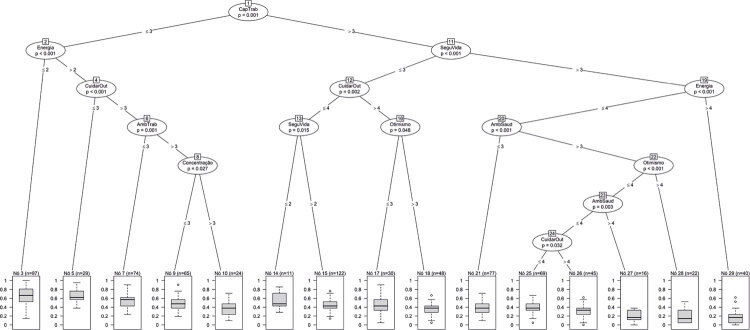
Árvore de classificação correspondendo ao desfecho de ‘despersonificação’, outra dimensão de burnout (Oldenburg Burnout Inventory). AmbSaud: ambiente saudável; AmbTrab: ambiente de trabalho; CuidarOut: cuidar de outras pessoas; CapTrab: capacidade para o trabalho; SeguVida: segurança de vida.

Na árvore de classificação correspondente ao desfecho de ‘despersonificação’ ( Figura 2 ), as respondentes representadas nas folhas 3 (n = 97, 12,6%) e 5 (n = 29, 3,8%) apresentaram os maiores escores de *burnout* , com média e mediana superior a 2,8 (correspondente a 0,6 na escala de 0 a 1). As 97 respondentes da folha 3 relataram insatisfação com a sua capacidade para o trabalho e pouca ou nenhuma energia para as tarefas diárias. No entanto, as 29 respondentes da folha 5, apesar de estarem insatisfeitas com a sua capacidade para o trabalho e não reconhecerem que cuidar de outras pessoas trouxesse sentido à vida, consideram ter energia suficiente para suas tarefas diárias.

**Figura 3:**
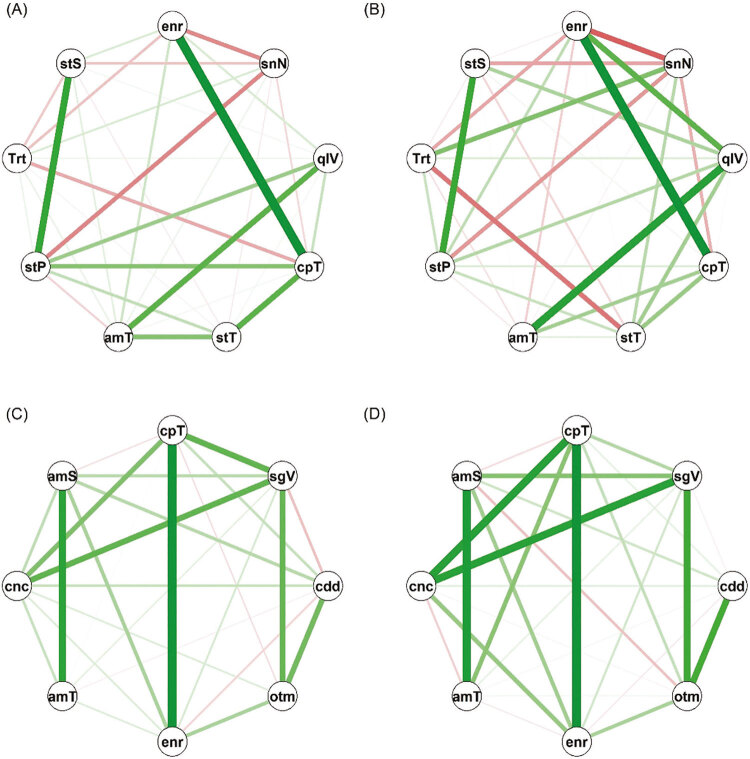
A matriz de correlação policórica é uma rede onde os nós são as variáveis, e os pesos nas arestas representam o coeficiente de correlação policórica. A espessura das arestas e a transparência são dadas pela magnitude do coeficiente de correlação entre os nós. Dimensões do burnout (Oldenburg Burnout Inventory): (A) Exaustão emocional sem burnout; (B) Exaustão emocional com burnout; (C) Despersonificação sem burnout; (D) Despersonificação com burnout. (*). Com burnout: amS: ambiente saudável; amT: ambiente de trabalho; cdd: cuidar de outras pessoas agrega sentido à vida; cnc: concentração; cpT: capacidade para o trabalho; enr: energia; otm: otimismo em momentos desafiadores; qlV - Qualidade de vida; sgV: segurança de vida; snN: sentimentos negativos; stP: satisfação com as relações pessoais; stS: satisfação com a vida sexual; stT: satisfação com o transporte; Trt: necessidade de tratamento médico. (*) Mais detalhes para compreender a matriz de correlação policórica estão disponíveis no material suplementar.

Para o desfecho de ‘exaustão emocional’ relacionado às mulheres médicas com *burnout* , o coeficiente de correlação policórica (Material Suplementar 3) identificou que ter sentimentos negativos teve correlação negativa com satisfação com a vida sexual e com as relações pessoais, bem como com energia para as tarefas diárias. A necessidade de tratamento médico para lidar com a vida diária correlacionou-se negativamente com a presença de energia para as tarefas diárias. No entanto, a falta de energia para as tarefas diárias correlacionou-se positivamente com a má qualidade de vida e com a insatisfação em relação à a capacidade para o trabalho ( Figura 3 ).

Para o desfecho de ‘despersonificação’ relacionado às mulheres médicas com *burnout* , o coeficiente de correlação policórica (Material Suplementar 3) identificou que a dificuldade de concentração foi correlacionada positivamente com a insatisfação em relação à capacidade para o trabalho e com a sensação de insegurança no dia a dia. A incapacidade de permanecer otimista em tempos de incerteza correlacionou-se positivamente com a sensação de insegurança no dia a dia e com a ausência de significado da própria vida no cuidado de outras pessoas. A falta de energia para as tarefas diárias correlacionou-se positivamente com a insatisfação em relação à capacidade para o trabalho ( Figura 3 ). Esses achados da análise de rede corroboram os da árvore de classificação e da regressão beta.

## Discussão

O presente estudo mostrou uma alta frequência de *burnout* entre as mulheres médicas brasileiras (61,6%) que responderam ao questionário. Em relação ao desfecho de ‘exaustão emocional’, as médicas com *burnout* tinham pouca ou nenhuma energia para as tarefas diárias, sentimentos negativos e insatisfação com a sua capacidade para o trabalho. Em relação ao desfecho de ‘despersonificação’, as médicas com *burnout* relataram insatisfação com a sua capacidade para o trabalho, pouca ou nenhuma energia para tarefas diárias e ausência de sentido à própria vida ao cuidar de outras pessoas, fatores que ameaçam a sua qualidade de vida. Apesar disso, apresentavam uma qualidade de vida relativamente boa e acreditavam que a espiritualidade as confortava e tranquilizava nos momentos difíceis.

O *burnout* tem sido definido como uma síndrome psicológica que resulta do estresse crônico no trabalho, suas principais dimensões sendo exaustão, cinismo e falta de eficácia profissional.^23^ A condição foi agravada pela pandemia de COVID-19 que desafia a saúde mental, questiona crenças pessoais e ameaça a qualidade de vida dos profissionais de saúde. O problema é exacerbado pelos afazeres domésticos tradicionalmente realizados pelas mulheres.^24^

O Brasil ocupou o segundo lugar em número de casos e óbitos por COVID-19. Isso levou o sistema de saúde brasileiro ao limite, afetando o atendimento aos pacientes não apenas com COVID-19, mas também com outras doenças agudas e crônicas. Quase todos os profissionais de saúde, principalmente médicos, estiveram envolvidos no combate à pandemia.^25^ Segundo Scheffer et al., o Brasil tem 477.982 médicos, sendo 222.942 mulheres, predominantemente jovens e residentes nas regiões Sudeste, Sul e Nordeste, a maioria (59,5%) com título de especialista. A medicina no Brasil está no processo de feminização.^26^ A distribuição das características da nossa amostra está de acordo com a relatada no estudo de Scheffer et al.

O nosso estudo mostrou maior prevalência de *burnout* (61,6%) entre as mulheres médicas brasileiras em comparação com outros estudos.^1 - 3 , 7 - 9^ O número crescente de casos suspeitos e confirmados de COVID-19, a grande carga de trabalho, a falta de equipamentos de proteção individual, a cobertura avassaladora da mídia, a falta de medicamentos específicos e a sensação de apoio insuficiente podem contribuir para o dano psicológico nos profissionais de saúde. Muitos temiam se infectar ou infectar suas famílias, amigos e colegas e relataram altos níveis de estresse, ansiedade e sintomas depressivos, que podem ter implicações psicológicas de longo prazo.^27 , 28^

Em nosso estudo, a maioria das respondentes relatou ter boa qualidade de vida e acreditar que sua vida tinha um propósito e que cuidar de outras pessoas trazia sentido à sua vida. Além disso, relataram ainda ter que superar barreiras no exercício da profissão e receber remuneração inferior à dos homens. Um grande número relatou sentimentos negativos frequentes, insatisfação com a vida sexual e falta de energia suficiente para as tarefas diárias, além de insatisfação com a aparência física. Tais achados podem ter sido influenciados pelos efeitos da pandemia em um país que vivencia enormes desafios no sistema de saúde.^25^ Esses resultados são semelhantes aos do estudo com médicos turcos, relatando que aqueles envolvidos na luta contra a COVID-19 relataram uma forte sensação de significância do trabalho.^29^ Porém, a frequência de *burnout* foi muito maior entre as médicas brasileiras, o que pode estar relacionado à magnitude dos efeitos da pandemia no Brasil.

A prática médica é permeada por experiências de perda, estresse, ansiedade e medo, que aumentam a vulnerabilidade psicológica dos médicos e facilitam o aparecimento de sintomas de ansiedade-depressão. Entretanto, resiliência, espiritualidade e crenças pessoais parecem desempenhar um papel mediador em algumas dessas variáveis psicológicas. Nenhum estudo avaliou como essas variáveis afetam a exaustão e o sofrimento psicológico de mulheres médicas, principalmente aquelas que atuam em um país que enfrenta um sistema de saúde deficitário.^30^ Observamos que as médicas confiam na espiritualidade para conforto e segurança, encontram força espiritual em tempos desafiadores e acreditam obter, pela fé, forças para os desafios diários.

Em nosso estudo, o uso inovador de aprendizado de máquina identificou, para o desfecho de ‘exaustão emocional’, que as mulheres médicas com *burnout* frequentemente têm pouca ou nenhuma energia para tarefas diárias e apresentam sentimentos negativos. Para o desfecho de ‘despersonificação’, as mulheres médicas relataram insatisfação com a sua capacidade para o trabalho, pouca ou nenhuma energia para as tarefas diárias e ausência de sentido à sua vida ao cuidar de outras pessoas ( Figuras 1 e 2 ). Até onde sabemos, não há avaliação de *burnout* entre mulheres médicas usando técnicas de inteligência artificial, que é o ponto forte do nosso estudo.

Além de confirmar os achados da regressão beta e das árvores de classificação em médicas com *burnout* , a análise de rede ( Figura 3 ) evidenciou a correlação de seus sentimentos negativos com insatisfação nas relações pessoais e na vida sexual, bem como com a falta de energia para tarefas diárias. Além disso, evidenciou a correlação da má qualidade de vida com a insatisfação em relação à capacidade para o trabalho e com a falta de energia para as tarefas diárias. Correlacionou ainda a dificuldade de concentração com a sensação de insegurança, que, por sua vez, foi correlacionada à incapacidade de permanecer otimista em tempos de incerteza. Além disso, esta análise mostrou graficamente a magnitude da relação entre as variáveis identificadas na técnica de aprendizado de máquina. Outro estudo que utilizou esse tipo de análise mostrou a associação da exaustão emocional elevada à hipertensão arterial e outras doenças crônicas,^6^ mesmo fora do período de pandemia.

Uma limitação do presente estudo é ter uma amostra de conveniência. A alta frequência de *burnout* pode ser devido a um viés amostral, isto é, mulheres com mais problemas podem ter respondido mais ao questionário do que outras mulheres. Apesar disso, a distribuição das características da amostra foi semelhante à observada no estudo de Demografia Médica no Brasil.^26^ O ponto forte do presente estudo é a análise conjunta usando aprendizado de máquina das condições de *burnout* , qualidade de vida e espiritualidade, bem como suas inter-relações durante a pandemia de COVID-19 em mulheres médicas que enfrentaram os desafios mais significativos ao lidar com condições únicas de trabalho e de vida, em um país assolado por casos e óbitos relacionadas à infecção por SARS-COV-2.

Os efeitos socioeconômicos da pandemia e como ela afetou um estilo de vida saudável, bem como o autocuidado feminino, a sensação de bem-estar e a qualidade de vida, representam grandes ameaças à saúde de mulheres médicas com *burnout* . No entanto, na maioria das vezes, tais assuntos não são reconhecidos nem abordados. Em países como o Brasil, que enfrenta muitos casos e óbitos, esse desafio é maior por conta das desigualdades de um país continental sem políticas direcionadas para a saúde dos médicos, principalmente para as mulheres médicas desafiadas pela tripla jornada de trabalho em tempos de pandemia.

Portanto, é essencial desenvolver estudos futuros para reconhecer a prevalência do *burnout* e seu impacto avassalador em diferentes populações para enfrentá-lo e preveni-lo adequadamente. Nossos achados destacam a importância de criar um ambiente propício à construção de relações de trabalho positivas. Adicionalmente, o governo e as agências de saúde devem fornecer recursos e investir para proteger o bem-estar psicológico de profissionais de saúde, criando programas de saúde mental. Paralelamente, devem ser estabelecidas parcerias com outras instituições sociais e implementados sistemas de atendimento remoto, movidos pela resiliência e compreensão de situações singulares, com a finalidade de auxiliar as médicas com *burnout* .

## Conclusão

O presente estudo mostrou uma alta frequência de *burnout* entre as médicas brasileiras que responderam ao questionário durante a pandemia de COVID-19. Apesar disso, apresentavam uma qualidade de vida relativamente boa e acreditavam que a espiritualidade trazia-lhes conforto e segurança nos momentos difíceis.

## * Material suplementar

Para informação adicional, por favor, clique aqui.



Para informação adicional do material suplementar 2, por favor, clique aqui.



Para informação adicional do material suplementar 3, por favor, clique aqui.


