# Supplementary material for: Mulheres Médicas: Burnout durante a Pandemia de COVID-19 no Brasil
Source: Arq Bras Cardiol. 2022 Jul 7;119(2):307–16. [Article in Portuguese] doi: 10.36660/abc.20210938 (PMC9363062; doi:10.36660/abc.20210938)
Supplement: Supplementary file 1 [file 2021-0938-Material-suplementar-dados-demograficos.pdf]

ETAPA 1: DADOS DEMOGRÁFICOS

1 Qual a sua idade?

Menos que 30 anos	1
De 31 a 40 anos	2
De 41 a 50 anos	3
De 51 a 60 anos	4
De 61 a 70 anos	5
Mais de 71 anos	6

2 Qual o seu estado conjugal

Nunca foi casado(a)	1
Casado(a) ou vive com companheiro(a)	2
Separado(a) ou divorciado(a)	3
Viúvo(a)	4

3 Quantos filhos você tem?

Não tenho filhos	1
1	2
2 ou 3	3
4 ou mais	4

4 Qual a sua cor?

Branca	1
Preta	2
Amarela	3
Parda	4

5 Qual a sua especialidade médica?

especialidades cirugicas	1
Especialidades clinicas	2
Cardiologia	3
Urgencia_CTI_Anestesia	4

6 Qual o estado em que mora:

Região Centro-Oeste	1
Região Nordeste	2
Região Norte	3
Região Sudeste	4
Região Sul	5

7 A localidade onde você trabalha é?

Uma grande cidade (acima de 500 mil habitantes)	1
Subúrbio ou arredores de uma grande cidade	2
Cidade de porte médio (entre 100 mil a 500 mil habitantes)	3
Pequena cidade (abaixo de 100 mil habitantes)	4

8 Exerce atualmente funções de direção, chefia ou coordenação?

Sim, formalmente	1
Sim, informalmente	2
Não	3

9 Em relação a estabilidade no emprego: Como você sente a sua situação no emprego atual:

Muito instável	1
Instável	2
Estável	3
Muito estável	4

10 Número de locais de trabalho

1	1
2	2
3	3
4 ou mais	4

11 Quantas horas por semana você dedica ao trabalho?

Até 20 horas por semana	1
De 21 a 36 horas por semana (por exemplo, 3 plantões de 12h por semana)	2

De 37 a 48 horas por semana (por exemplo, 4 plantões de 12h por semana)	3
De 49 a 60 horas por semana (por exemplo, 5 plantões de 12h por semana)	4
Mais de 61 horas por semana (por exemplo, mais de 5 plantões de 12h por semana)	5

12 Quanto tempo você dedica a atividades domésticas?

Até 5 horas por semana	1
De 6 a 10 horas por semana	2
De 11 a 20 horas por semana	3
Mais de 21 horas por semana	4

13 Quantas horas por semana você dedicada ao lazer?

Até 5 horas por semana	1
De 6 a 10 horas por semana	2
De 11 a 20 horas por semana	3
Mais de 21 horas por semana	4

14 Qual é a sua faixa salarial?

Menos de 2.500,00	1
De 2.501,00 a 5.000,00	2
De 5.001,00 a 10.000,00	3
De 10.001,00 a 20.000,00	4
Mais de 20.001,00	5

15 Com relação à minha RENDA (pessoal + familiar) e a pandemia de COVID-19:

Não houve alteração em minha renda.	1
Houve redução de cerca de até 20% de minha renda.	2
Houve redução de cerca de 21 a 50% em minha renda.	3
Houve redução de 50% ou mais em minha renda	4
Houve aumento de minha renda.	5

16 Como você considera as suas condições de trabalho, aqui interpretado como ambiente de trabalho?

Péssima	1
Ruim	2
Regular	3
Boa	4
Excelente	5

17 Como você considera as condições e disponibilidade dos EPIs no seu trabalho?

Péssima	1
Ruim	2
Regular	3
Boa	4
Excelente	5

18 Ao longo da sua formação você teve que interrompê-la por motivos familiares/pessoais? Por quanto tempo?

Não interrompi	1
1-2 meses	2
3-6 meses	3
6-11 meses	4
12 meses ou mais	5

19 Você sofre ou já sofreu assédio moral durante a sua formação e/ou no exercício da medicina?

Não	1
Sim, porém foi pontual	2
Sim, com pouca frequência	3
Sim, com muita frequência	4

20 Se você já sofreu assédio moral (respondeu sim na pergunta anterior) você buscou ajuda? Obteve auxílio?

Nunca sofri assédio moral	1
Sofri assédio moral, mas não procurei ajuda	2
Sofri assédio moral, procurei ajuda e não obtive	3
Sofri assédio moral, procurei ajuda e obtive o auxílio esperado	4

21 Você já se sentiu discriminada pelo fato de ser mulher no seu ambiente de trabalho, durante o exercício da medicina?

Não	1
Sim, com pouca frequência	2
Sim, com muita frequência	3

Etapa 2: QUALIDADE DE VIDA

22 Como você avaliaria sua qualidade de vida?

Muito Ruim	1
Ruim	2
Nem ruim nem boa	3
Boa	4
Muito boa	5

23 Quão satisfeito(a) você está com a sua saúde?

Muito satisfeito	1
Insatisfeito	2
Nem satisfeito nem insatisfeito	3
Satisfeito	4
Muito insatisfeito	5

24 Em que medida você acha que sua dor (física) impede você de fazer o que você precisa?

Nada	1
Muito Pouco	2
Mais ou menos	3
Bastante	4
Extremamente	5

25 O quanto você precisa de algum tratamento médico para levar sua vida diária?

Nada	1
Muito Pouco	2
Mais ou menos	3
Bastante	4
Extremamente	5

26 O quanto você aproveita a vida?

Nada	1
Muito pouco	2
Mais ou menos	3
Bastante	4
Extremamente	5

27 Em que medida você acha que a sua vida tem sentido?

Nada	1
Muito Pouco	2
Mais ou menos	3
Bastante	4
Extremamente	5

28 O quanto você consegue se concentrar?

Nada	1
Muito Pouco	2
Mais ou menos	3
Bastante	4
Extremamente	5

29 Quão seguro(a) você se sente em sua vida diária?

Nada	1
Muito Pouco	2
Mais ou menos	3
Bastante	4
Extremamente	5

30 Quão saudável é o seu ambiente físico (clima, barulho, poluição, atrativos)?

Nada	1
Muito Pouco	2
Mais ou menos	3
Bastante	4
Extremamente	5

31 Você tem energia suficiente para seu dia-a-dia?

Nada	1
Muito Pouco	2
Mais ou menos	3
Bastante	4
Completamente	5

32 Você aceita a sua aparência física?

Nada	1
Muito Pouco	2
Mais ou menos	3
Bastante	4
Completamente	5

33 Você ganha o suficiente para satisfazer suas necessidades?

Nada	1
Muito Pouco	2
Mais ou menos	3
Bastante	4
Completamente	5

34 Quão disponíveis para você estão as informações que precisa no seu dia-a-dia?

Muito Pouco	1
Mais ou menos	2
Bastante	3
Completamente	4

35 Em que medida você tem oportunidades de atividade de lazer?

Nada	1
Muito Pouco	2
Mais ou menos	3
Bastante	4
Completamente	5

36 Quão satisfeito você está com sua capacidade de se locomover?

Muito insatisfeito	1
Insatisfeito	2
Nem satisfeito nem insatisfeito	3
Satisfeito	4
Muito satisfeito	5

37 Quão satisfeito(a) você está com o seu sono?

Muito insatisfeito	1
Insatisfeito	2
Nem satisfeito nem insatisfeito	3
Satisfeito	4
Muito satisfeito	5

38 Quão satisfeito(a) você está com sua capacidade de desempenhar as atividades do seu dia-a-dia?

Muito insatisfeito	1
Insatisfeito	2
Nem satisfeito nem insatisfeito	3
Satisfeito	4
Muito satisfeito	5

39 Quão satisfeito(a) você está com sua capacidade para o trabalho?

Muito insatisfeito	1
Insatisfeito	2
Nem satisfeito nem insatisfeito	3
Satisfeito	4
Muito satisfeito	5

40 Quão satisfeito(a) você está consigo mesmo?

Muito insatisfeito	1
Insatisfeito	2
Nem satisfeito nem insatisfeito	3
Satisfeito	4
Muito satisfeito	5

41 Quão satisfeito(a) você está com suas relações pessoais (amigos, parentes, conhecidos, colegas)?

Muito insatisfeito	1
Insatisfeito	2
Nem satisfeito nem insatisfeito	3
Satisfeito	4
Muito satisfeito	5

42 Quão satisfeito(a) você está com sua vida sexual?

Muito insatisfeito	1
Insatisfeito	2
Nem satisfeito nem insatisfeito	3
Satisfeito	4
Muito satisfeito	5

43 Quão satisfeito(a) você está com o apoio que você recebe de seus amigos?

Muito insatisfeito	1
Insatisfeito	2
Nem satisfeito nem insatisfeito	3
Satisfeito	4
Muito satisfeito	5

44 Quão satisfeito(a) você está com as condições do local onde mora?

Muito insatisfeito	1
Insatisfeito	2
Nem satisfeito nem insatisfeito	3
Satisfeito	4
Muito satisfeito	5

45 Quão satisfeito(a) você está com o seu acesso aos serviços de saúde?

Muito insatisfeito	1
Insatisfeito	2
Nem satisfeito nem insatisfeito	3
Satisfeito	4
Muito satisfeito	5

46 Quão satisfeito(a) você está com o seu meio de transporte?

Muito insatisfeito	1
Insatisfeito	2
Nem satisfeito nem insatisfeito	3
Satisfeito	4
Muito satisfeito	5

47 Com que frequência você tem sentimentos negativos tais como, mau humor, desespero, ansiedade, depressão?

Nunca	1
Algumas vezes	2
Frequentemente	3
Muito frequentemente	4
Sempre	5

Etapa 3 - QUALIDADE DE VIDA – MÓDULO ESPIRITUALIDADE, RELIGIÃO E CRENÇAS PESSOAIS

48 Até que ponto alguma ligação com o espiritual conforta/tranquiliza você?

Nada	1
Muito Pouco	2
Mais ou menos	3
Bastante	4
Extremamente	5

49 Até que ponto cuidar de outras pessoas proporciona um sentido na vida para você?

Nada	1
Muito Pouco	2
Mais ou menos	3
Bastante	4
Extremamente	5

50 Até que ponto você consegue ter admiração pelas coisas a seu redor? (por exemplo: natureza, arte, música)

Muito Pouco	1
Mais ou menos	2
Bastante	3
Extremamente	4

51 Até que ponto você sente alguma ligação entre a sua mente, corpo e alma?

Nada	1
Muito Pouco	2
Mais ou menos	3
Bastante	4
Extremamente	5

52 Até que ponto você pode encontrar força espiritual em épocas difíceis?

Nada	1
Muito Pouco	2
Mais ou menos	3
Bastante	4
Extremamente	5

53 Até que ponto você tem paz interior?

Nada	1
Muito Pouco	2
Mais ou menos	3
Bastante	4
Extremamente	5

54 Quanto você é capaz de permanecer otimista em épocas de incerteza?

Nada	1
Muito Pouco	2
Mais ou menos	3
Bastante	4
Extremamente	5

55 Até que ponto a fé lhe dá força no dia-a-dia?

Nada	1
Muito Pouco	2
Mais ou menos	3
Bastante	4
Extremamente	5

56 Até que ponto a participação em uma comunidade religiosa ou espiritual lhe dá suporte?

Nada	1
Muito Pouco	2
Mais ou menos	3
Bastante	4
Extremamente	5

Etapa 4: BURNOUT

57 Há dias em que me sinto cansado ainda antes mesmo de chegar ao trabalho:

Discordo completamente	1
Discordo	2
Concordo	3
Concordo completamente	4

58 Depois do trabalho, preciso de mais tempo para relaxar e sentir-me melhor do que precisava antigamente:

Discordo completamente	1
Discordo	2
Concordo	3
Concordo completamente	4

59 Consigo suportar muito bem as pressões do meu trabalho:

Discordo completamente	4
Discordo	3
Concordo	2
Concordo completamente	1

60	Durante o meu trabalho, sinto-me emocionalmente esgotado:	
	Discordo completamente	1
	Discordo	2
	Concordo	3
	Concordo completamente	4
61	Depois das tarefas profissionais, tenho geralmente energia para as minhas atividades de lazer:	
	Discordo completamente	4
	Discordo	3
	Concordo	2
	Concordo completamente	1
62	Depois do meu trabalho, sinto-me cansado e sem energia:	
	Discordo completamente	1
	Discordo	2
	Concordo	3
	Concordo completamente	4
63	Com frequência faço coisas novas e interessantes no meu trabalho:	
	Discordo completamente	4
	Discordo	3
	Concordo	2
	Concordo completamente	1
64	Cada vez falo mais e com mais frequência de forma negativa sobre meu trabalho:	
	Discordo completamente	1
	Discordo	2
	Concordo	3
	Concordo completamente	4
65	Ultimamente, tenho realizado meu trabalho de forma quase mecânica:	
	Discordo completamente	1
	Discordo	2
	Concordo	3
	Concordo completamente	4
66	Considero meu trabalho um desafio positivo:	
	Discordo completamente	4
	Discordo	3
	Concordo	2
	Concordo completamente	1
67	Com o passar do tempo, venho me desinteressando do meu trabalho:	
	Discordo completamente	1
	Discordo	2
	Concordo	3
	Concordo completamente	4
68	Sinto-me cada vez mais empenhado no meu trabalho:	
	Discordo completamente	4
	Discordo	3
	Concordo	2
	Concordo completamente	1
69	Muitas vezes, sinto-me farto das minhas tarefas	
	Discordo completamente	1
	Discordo	2
	Concordo	3
	Concordo completamente	4
