# Supplementary material for: Mulheres Médicas: Burnout durante a Pandemia de COVID-19 no Brasil
Source: Arq Bras Cardiol. 2022 Jul 7;119(2):307–16. [Article in Portuguese] doi: 10.36660/abc.20210938 (PMC9363062; doi:10.36660/abc.20210938)
Supplement: Supplementary file 2 [file 2021-0938-Material-suplementar-extra.pdf]

Supplementary Material:

Independent variables

The cutoff points of the independent variables were defined according to the classification tree corresponding to each outcome.

For the outcome emotional exhaustion, the following values were considered:

**Independent variable**	**Cutoff point**
31 Energy	≤ 3 and > 3
47 NegFeel	≤ 2 and > 2
22 QoL	≤ 3 and > 3
39 CapWork	≤ 2 and > 2
46 TranspSatisf	≤ 2 and > 2
16 WorkEnvir	≤ 3 and > 3
41 PersSatisf	≤ 3 and > 3
25 NeedTreat	≤ 2 and > 2
42 SexSatisf	≤ 3 and > 3

NegFeel: negative feelings
QoL: quality of life
CapWork: capability for work
TranspSatisf: satisfaction with transportation
WorkEnvir: work environment
PerspSatisf: satisfaction with personal relations
NeedTreat: need for treatment
SexSatisf: satisfaction with sexual life

For the outcome disengagement, the following values were considered:

**Independent variable**	**Cutoff point**
39 CapWork	≤ 3 and > 3
29 LifeSafe	≤ 3 and > 3

49 CareOthers	≤ 3 and > 3
54 Optimism	≤ 3 and > 3
31 Energy	≤ 3 and > 3
16 WorkEnvir	≤ 3 and > 3
28 Concentration	≤ 3 and > 3
30 HealthyEnvir	≤ 3 and > 3

CapWork: capability for work
LifeSafe: life safety
CareOthers: caring for others
WorkEnvir: work environment
HealthyEnvir: healthy environment

Outcomes

The cutoff points of the dependent variables were defined according to two classification trees: the first tree was implemented considering 'disengagement' as outcome and 'emotional exhaustion' as independent variable (Figure 1); the second tree was implemented considering 'emotional exhaustion' as outcome and 'disengagement' as independent variable (Figure 2).

The cutoff point for each outcome was defined according to the cutoff point observed for the independent variable. The following values were considered:

**Dependent variable**	**Cutoff point**
Emotional exhaustion	≤ 2.668 and > 2.668 (= 0.556 scale of 0 to 1)
Disengagement	≤ 2.143 and > 2.143 (= 0.381 scale of 0 to 1)

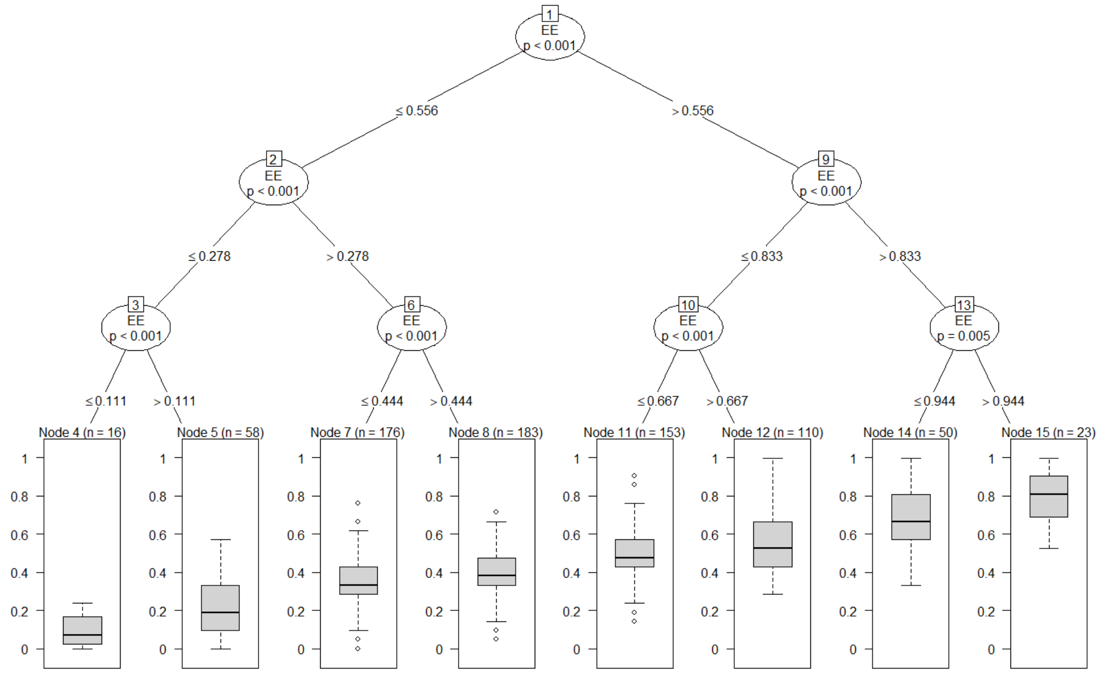

Figure 1. Classification tree considering ‘disengagement’ as outcome and ‘emotional exhaustion’ as the independent variable. EE: emotional exhaustion

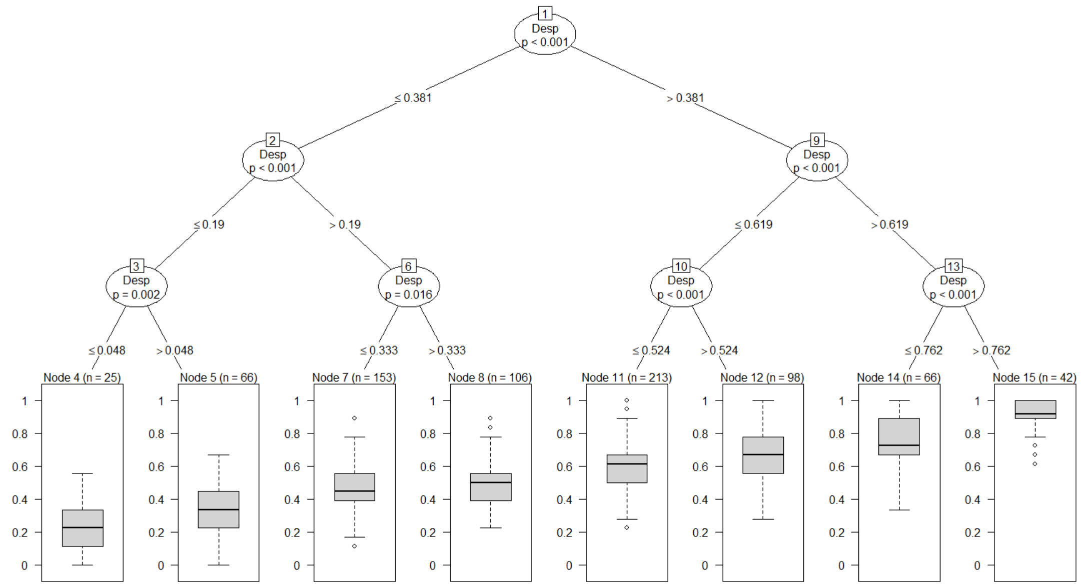

Figure 2. Classification tree considering ‘emotional exhaustion’ as outcome and ‘disengagement’ as the independent variable. Diseng: disengagement
