# Supplementary material for: Mulheres Médicas: Burnout durante a Pandemia de COVID-19 no Brasil
Source: Arq Bras Cardiol. 2022 Jul 7;119(2):307–16. [Article in Portuguese] doi: 10.36660/abc.20210938 (PMC9363062; doi:10.36660/abc.20210938)
Supplement: Supplementary file 3 [file 2021-0938-Material-suplementar-burnout.pdf]

Exaustão Emocional – sem burnout

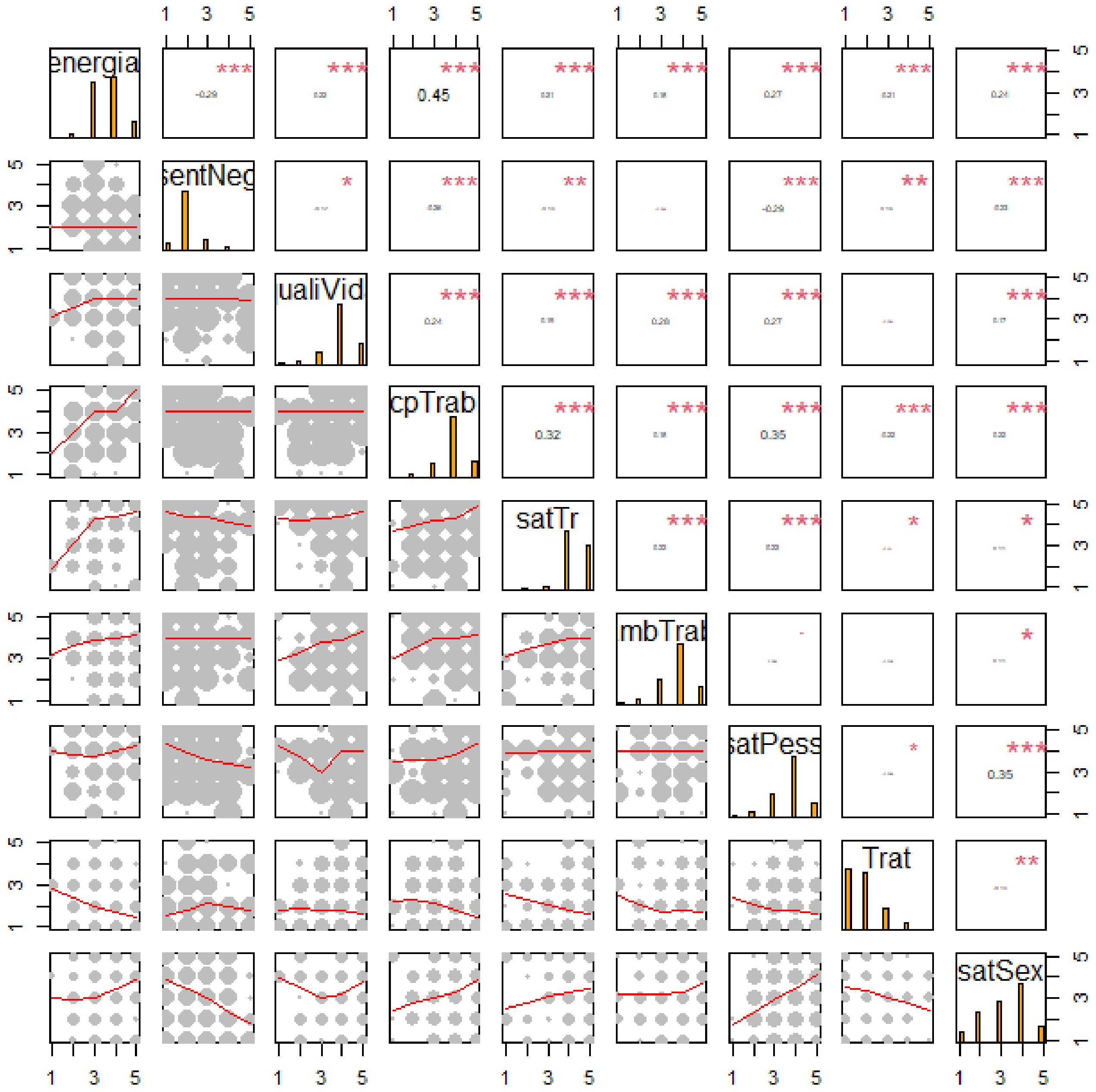

	energia	sentNeg	qualiVida	cpTrab	satTr	ambTrab	satPess	Trat	satSex
energia	1.00	-0.37	0.28	0.57	0.29	0.23	0.32	-0.26	0.27
sentNeg	-0.37	1.00	-0.16	-0.35	-0.21	-0.07	-0.37	0.20	-0.28
qualiVida	0.28	-0.16	1.00	0.32	0.24	0.33	0.31	-0.06	0.20
cpTrab	0.57	-0.35	0.32	1.00	0.44	0.23	0.43	-0.28	0.26
satTr	0.29	-0.21	0.24	0.44	1.00	0.29	0.30	-0.14	0.14
ambTrab	0.23	-0.07	0.33	0.23	0.29	1.00	0.11	-0.03	0.13
satPess	0.32	-0.37	0.31	0.43	0.30	0.11	1.00	-0.14	0.42
Trat	-0.26	0.20	-0.06	-0.28	-0.14	-0.03	-0.14	1.00	-0.18
satSex	0.27	-0.28	0.20	0.26	0.14	0.13	0.42	-0.18	1.00

Exaustão Emocional – com burnout

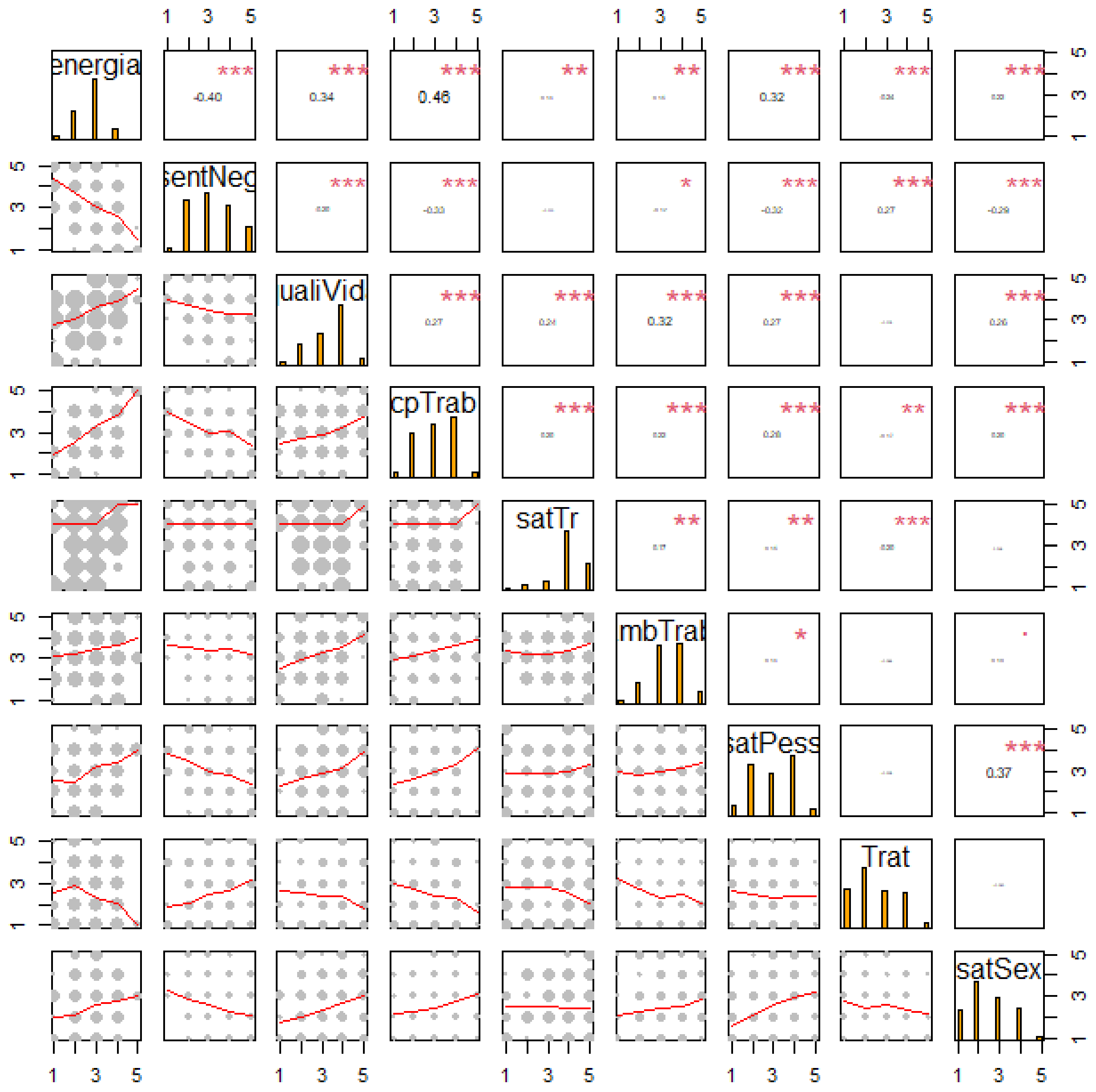

	energia	sentNeg	qualiVida	cpTrab	satTr	ambTrab	satPess	Trat	satSex
energia	1.00	-0.47	0.41	0.56	0.19	0.17	0.37	-0.28	0.23
sentNeg	-0.47	1.00	-0.25	-0.38	-0.06	-0.14	-0.36	0.29	-0.32
qualiVida	0.41	-0.25	1.00	0.32	0.26	0.39	0.34	-0.11	0.28
cpTrab	0.56	-0.38	0.32	1.00	0.28	0.26	0.32	-0.22	0.21
satTr	0.19	-0.06	0.26	0.28	1.00	0.19	0.17	-0.23	0.04
ambTrab	0.17	-0.14	0.39	0.26	0.19	1.00	0.12	-0.12	0.10
satPess	0.37	-0.36	0.34	0.32	0.17	0.12	1.00	-0.07	0.42
Trat	-0.28	0.29	-0.11	-0.22	-0.23	-0.12	-0.07	1.00	-0.10
satSex	0.23	-0.32	0.28	0.21	0.04	0.10	0.42	-0.10	1.00

Despersonificação – sem burnout

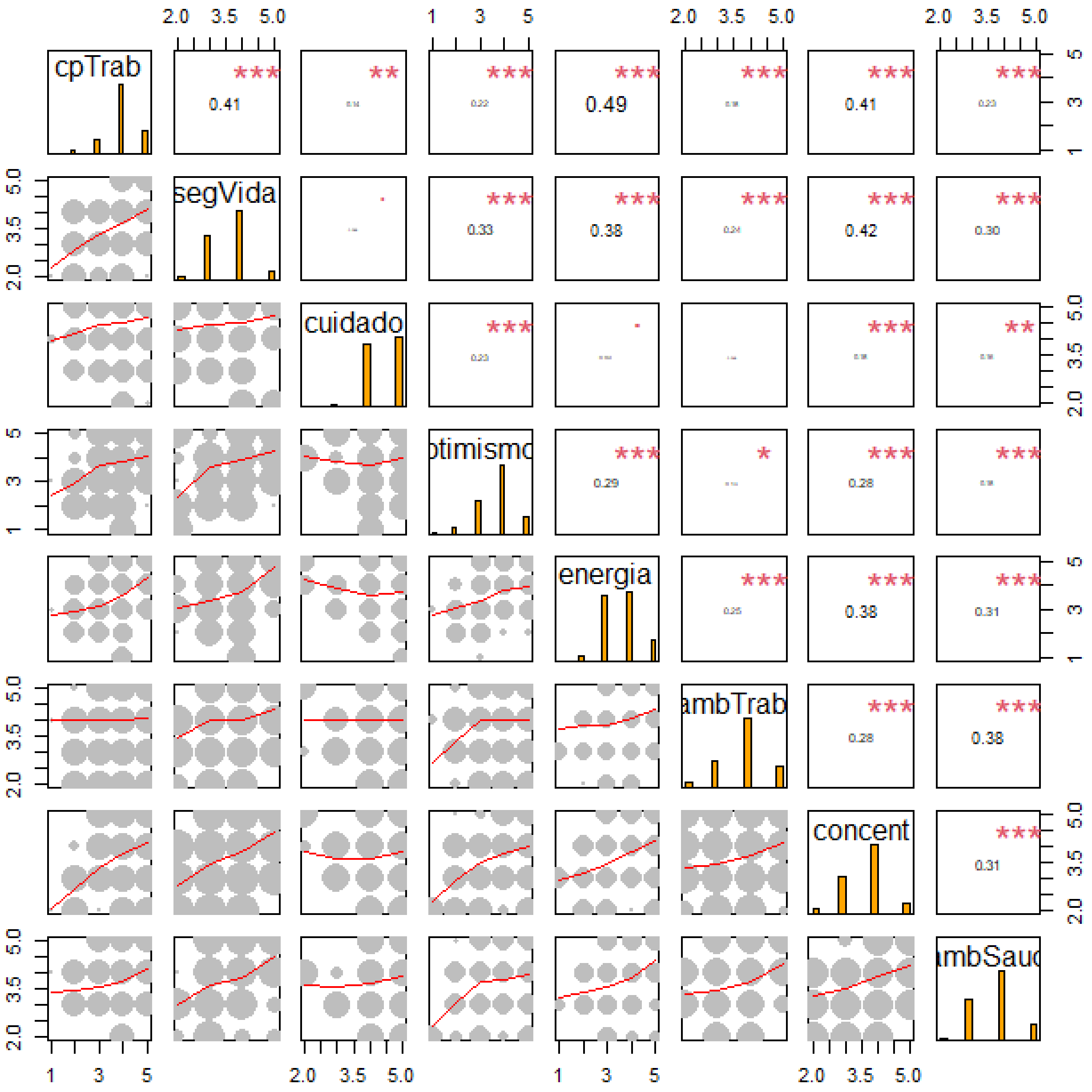

	cpTrab	segVida	cuidado	otimismo	energia	ambTrab	concent	ambSaud
cpTrab	1.00	0.54	0.19	0.28	0.59	0.23	0.52	0.28
segVida	0.54	1.00	0.12	0.42	0.48	0.30	0.55	0.38
cuidado	0.19	0.12	1.00	0.29	0.12	0.09	0.23	0.21
otimismo	0.28	0.42	0.29	1.00	0.35	0.18	0.34	0.24
energia	0.59	0.48	0.12	0.35	1.00	0.29	0.45	0.37
ambTrab	0.23	0.30	0.09	0.18	0.29	1.00	0.33	0.46
concent	0.52	0.55	0.23	0.34	0.45	0.33	1.00	0.39
ambSaud	0.28	0.38	0.21	0.24	0.37	0.46	0.39	1.00

Despersonificação – com burnout

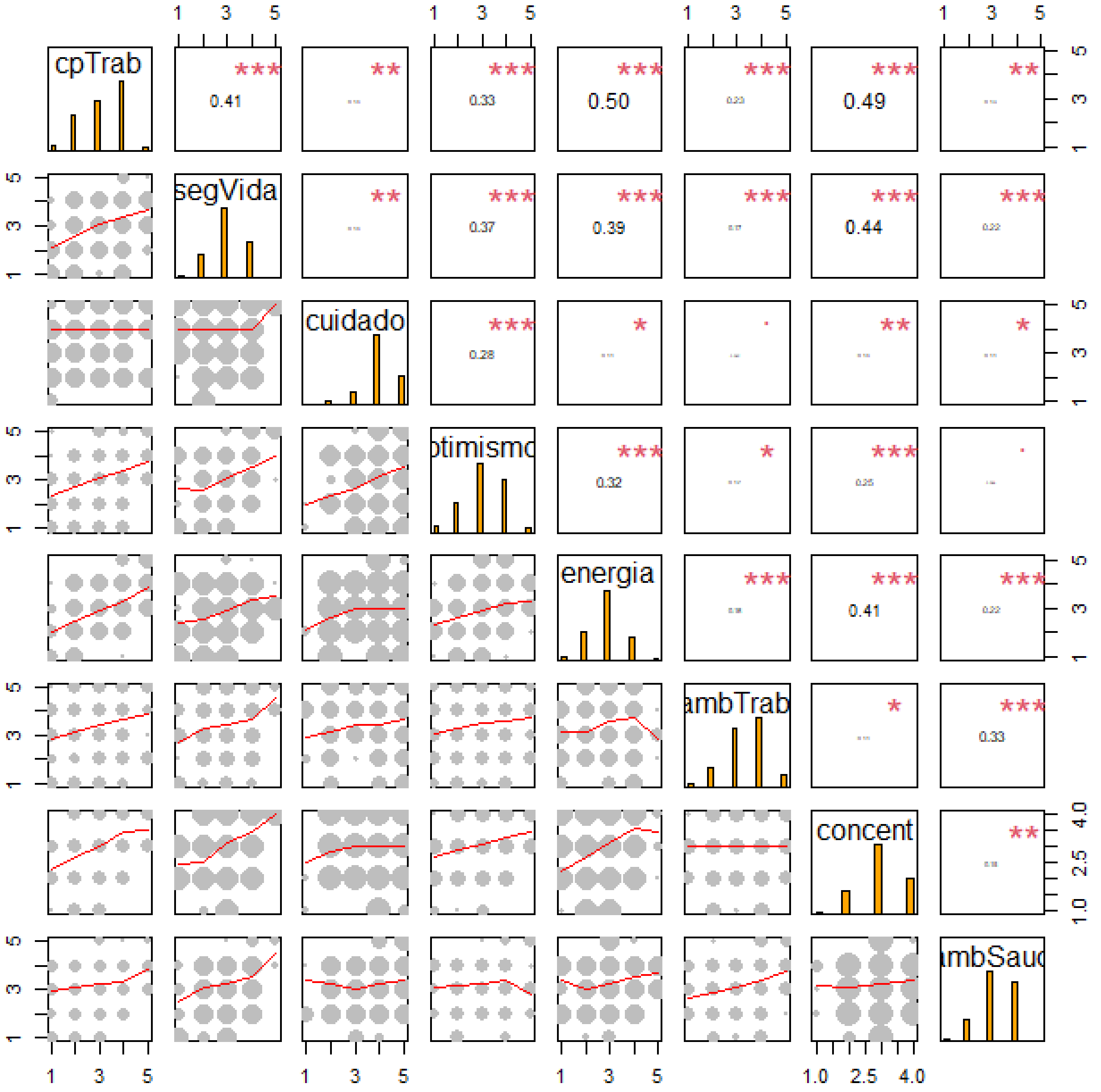

	cpTrab	segVida	cuidado	otimismo	energia	ambTrab	concent	ambSaud
cpTrab	1.00	0.49	0.19	0.38	0.59	0.26	0.56	0.20
segVida	0.49	1.00	0.17	0.44	0.45	0.22	0.53	0.30
cuidado	0.19	0.17	1.00	0.32	0.14	0.11	0.17	0.13
otimismo	0.38	0.44	0.32	1.00	0.36	0.14	0.30	0.10
energia	0.59	0.45	0.14	0.36	1.00	0.17	0.49	0.26
ambTrab	0.26	0.22	0.11	0.14	0.17	1.00	0.12	0.38
concent	0.56	0.53	0.17	0.30	0.49	0.12	1.00	0.18
ambSaud	0.20	0.30	0.13	0.10	0.26	0.38	0.18	1.00
