# Supplementary material for: Mulheres Médicas: Burnout durante a Pandemia de COVID-19 no Brasil
Source: Arq Bras Cardiol. 2022 Jul 7;119(2):307–16. [Article in Portuguese] doi: 10.36660/abc.20210938 (PMC9363062; doi:10.36660/abc.20210938)
Supplement: Translation

# Translation: Women Physicians: Burnout during the COVID-19 Pandemic in Brazil

**Authors:** Gláucia Maria Moraes de Oliveira, Viviana Guzzo Lemke, Maria Sanali Moura de Oliveira Paiva, Giordana Zeferino Mariano, Elizabeth Regina Giunco Alexandre Silva, Sheyla Cristina Tonheiro Ferro da Silva, Magaly Arrais dos Santos, Imara Correia de Queiroz Barbosa, Carla Janice Baister Lantieri, Elizabeth da Rosa Duarte, Maria Cristina Oliveira Izar, Karin Jaeger Anzolch, Milena Alonso Egea Gerez, Mayara Viana de Oliveira Ramos, Maria Antonieta Albanez Albuquerque de Medeiro Lopes, Emilia Matos do Nascimento, Nanette Kass Wenger

**Affiliations:** 1 Brazil Universidade Federal do Rio de Janeiro, Rio de Janeiro, RJ – Brazil; 2 Brazil Cardiocare, Curitiba, PR – Brazil; 3 Brazil Hospital Universitário Onofre Lopes, Natal, RN – Brazil; 4 Brazil Hospital São João Batista, Criciúma, SC – Brazil; 5 Brazil Hospital do Coração, São Paulo, SP – Brazil; 6 Brazil CEMISE, Aracaju, SE – Brazil; 7 Brazil Universidade Federal de Campina Grande, Campina Grande, PB – Brazil; 8 Brazil Centro Universitário Faculdade de Medicina do ABC, Santo André, SP – Brazil; 9 Brazil Hospital Nossa Senhora da Conceição, Porto Alegre, RS – Brazil; 10 Brazil Universidade Federal de São Paulo, São Paulo, SP – Brazil; 11 Brazil Hospital Moinhos de Vento, Porto Alegre, RS – Brazil; 12 Brazil Instituto de Moléstias Cardiovasculares, São José do Rio Preto, SP – Brazil; 13 Brazil Universidade Federal do Maranhão, São Luis, MA – Brazil; 14 Brazil Real Hospital Português, Recife, PE – Brazil; 15 Brazl Universidade do Estado do Rio de Janeiro, Rio de Janeiro, RJ – Brazl; 16 USA Emory University School of Medicine Atlanta, Georgia – USA

**Keywords:** Physicians, Women, Burnout, Psychological, COVID-19, Brazil

## Abstract

**Background:**

COVID-19 has placed a tremendous burden on physicians worldwide, especially women physicians, affected by increased workload and loss of quality of life.

**Objective:**

To assess the effects of the COVID-19 pandemic on the quality of life, burnout and spirituality of Brazilian women physicians directly or indirectly providing care to COVID-19 patients.

**Methods:**

Prospective, observational study performed from July 28 to September 27, 2020, in Brazil, with women physicians from 47 specialities, the most frequent being cardiology (22.8%), with no age restriction. They voluntarily answered an online survey with questions on demographic and socioeconomic characteristics, quality of life (WHOQOL-brief), spirituality (WHOQOL-SRPB), and statements from the Oldenburg Burnout Inventory. Statistical analysis used the R software, beta regression, classification trees, and polychoric correlation matrix, with a 5% of significance level.

**Results:**

Of the 769 respondents, 61.6% reported signs of burnout. About 64% reported wage loss of up to 50% during the pandemic. Some reported lack of energy for daily tasks, frequent negative feelings, dissatisfaction with capability for work, and caring for others not adding meaning to their lives. Negative feelings correlated negatively with satisfaction with sexual life and personal relations, and energy for daily tasks. The inability to remain optimistic in times of uncertainty correlated positively with feeling unsafe daily and not acknowledging that caring for others brings meaning to life.

**Conclusion:**

This study showed a high frequency of burnout among Brazilian women physicians who answered the survey during the COVID-19 pandemic. Nevertheless, they presented with a relatively good quality of life and believed that spirituality comforted and reassured them in hard times.

## Introduction

Physicians on the front lines against the coronavirus disease-2019 (COVID-19) have faced unprecedented high levels of stress. Nevertheless, little attention has been paid to the vulnerability experienced by these professionals, mainly of the female sex. A systematic review carried out in the database Medline and Embase has shown an increase in the challenges related to the high workload and loss of quality of life during the COVID-19 pandemic, which are associated with physical and mental exhaustion.^1^ The prevalence of burnout ranged from 23% to 76%, and female gender, high workload, and family-related concerns are predictors of burnout.^1^ The authors recommended that studies on physician burnout take gender differences into account.

A study, conducting a cross-sectional survey to assess 2707 healthcare professionals (HCPs) from 60 countries, reported that 51% of them had burnout, which was associated with work impacting household activities, exposure to COVID-19 patients, inadequate training, and making life prioritizing decisions. Burnout was more frequent in high-income countries.^2^ Another study reported an increase in burnout in women physicians as compared to men physicians, and the authors hypothesized that specific stressors included shortage of daycare options for children and imbalance between work and personal life. Women physicians with greater workloads and those without a partner experienced higher levels of burnout.^3^ It is worth noting that women currently constitute a great proportion of the global health workforce and spend 15 hours more per week on unpaid domestic labor.^4 , 5^

The burnout dimensions have been significantly associated with an increased risk for diseases, independently of sociodemographic factors and depressive symptoms. A study with 5671 participants [predominantly physicians, mean age of 44.1 years (range, 18-70 years), 62.4% women] used a mobile health web application for an online survey of job burnout measured with the Maslach Burnout Inventory-General Survey. By using network analysis and logistic regression, the study has shown the association of high emotional exhaustion with arterial hypertension and other chronic diseases after adjusting for age, sex, educational level, and depressive symptoms.^6^

Another systematic review with 12 studies assessing burnout in HCPs working or not in frontline COVID-19 wards has shown controversial results.^7^ Two of the studies reported higher levels of emotional fatigue in women as compared to men and that female sex was a risk factor for burnout among intensive care professionals.^8 , 9^ However, another study has found no association with gender.^10^ The heterogeneity of the studies regarding data collection and questionnaires used could have accounted for that, which emphasizes the need for further studies.^7^

Brazil has ranked second in number of COVID-19 cases and deaths since the beginning of the pandemic. However, to our knowledge, no study has assessed the burnout of Brazilian women physicians during the pandemic. Thus, this study aimed to assess the effects of the pandemic on the quality of life, burnout, and spirituality of women physicians directly or indirectly providing care to COVID-19 patients.

## Methods

This is a transversal, observational study performed from July 28 to September 27, 2020 in Brazil, with women physicians from different medical specialities, directly or indirectly providing care to COVID-19 patients. There was no age restriction. The women physicians voluntarily answered an online survey with 68 questions, thus constituting a convenience sample.

The survey consisted of the following: 20 questions on demographic and socioeconomic characteristics; the 26 questions from the Brazilian Portuguese version of the WHOQOL-brief;^11^ 9 questions based on the World Health Organization Quality of Life Spirituality, Religiousness and Personal Beliefs (WHOQOL-SRPB) field-test instrument;^12 , 13^ and the 13 statements from the Brazilian Portuguese version of the Oldenburg Burnout Inventory (OLBI)^14 - 16^ (Supplementary Material 1).

Through user identification, participants who answered the survey multiple times could be identified. All participants provided informed consent for the use of their anonymized data.

This study was approved by the Ethics Committee on Research (HUOL-CAAE: 34673520.7.0000.5292).

According to the methodology proposed by Schuster et al. and Demerouti et al., the 13 burnout statements of the OLBI were transformed into variables of the two dimensions ‘disengagement’ (7 variables) and ‘emotional exhaustion’ (6 variables).^15 , 17^ Questions which answer was agree or disagree had the score inverted so as to the higher the score of each variable, the higher the burnout level. Each dimension was attributed a score corresponding to its mean score.

### Statistical Analysis

Statistical analysis was performed through Beta Regression^18^ which models rates and proportions outcomes. The two burnout dimensions were considered as outcomes and the 55 remaining questions, as independent variables.

In the OLBI, each outcome’s score is limited to the 1-4 interval, thus a beta regression model was implemented, where each outcome was recalculated by using linear interpolation, so that values from 0 to 1 could be obtained. Three models were implemented for each outcome. The first model was composed of 55 variables. The second and third models used the independent variables that showed 10% significance in the previous model.

After the beta regression models, regression trees were implemented using the final model’s independent variables and their respective outcomes. The Classification and Regression Trees (CART) is a nonparametric method used to obtain an association between the dependent variable and a set of covariates. Decision trees are used to identify the interaction between covariates. The leaves of the tree provide a graphical representation of the outcome for each group of individuals. The betareg^18^ and partykit packages in R were used to implement the regression tree’s beta regression models.^19 , 20^

Another graphical visualization was used based on a polychoric correlation,^21^ a measure of association between ordinal categorical variables. A polychoric correlation matrix was represented as a network where the nodes were the variables, and the weights on the edges represented the polychoric correlation coefficient. The thickness of the edges and the transparency was given by the magnitude of the correlation coefficient between the nodes. The colors red and green corresponded to negative and positive correlations, respectively. The ‘qgraph’ package in R was used for network visualization.^20 - 22^

For the statistical tests, we adopted a significance level of 5%.

## Results

Of the 769 respondents, 474 (61.6%) reported signs of burnout. The criterion for classification of the respondents was provided by the cutoff points obtained from the classification trees: emotional exhaustion (<2.668 and ≥2.668) and disengagement (<2.143 and ≥2.143) (Supplementary Material 2).

Based on the answers to the questions, the characteristics of the sample were as follows: under 50 years of age, 50.2%; white skin color, 81.9%; married, 87.8%; and with 1 to 3 children, 67.5%. The distribution of the 47 medical specialities was as follows: Cardiology, 22.8%; Pediatrics, 15%; Internal Medicine, 6%; Obstetrics and Gynecology, 5.6%; Anesthesiology, 3.8%; Family and Community Medicine, 2.9%; and Intensive Care Medicine, 2.5%. All five Brazilian geographic regions were represented, the most frequent being the Southeastern (34.3%), Southern (31.7%), and Northeastern (28.3%) regions.

Most respondents worked in cities with more than 500 000 inhabitants (74.1%), were not in a leadership position (66.2%), had work stability (74.5%), and worked in two or three different places (59.7%). They spent 6 to 20 hours per week with household chores (54.8%) and up to 5 hours with leisure activities (59.0%). About 64% of the respondents earned US$ 1000 to US$ 4000, and 57.6% reported wage loss of as much as 50% during the pandemic. They reported good work conditions (61%) and availability of proper personal protective equipment (61.5%).

Most respondents reported having a good quality of life (71.7%) and being satisfied with their health (55%), while 64.8% reported not really enjoying life, almost 80% reported believing their lives had a purpose and 90.4% acknowledged that caring for others brought meaning to their lives. They considered the following aspects of their lives satisfactory: sleep, 62.9%; ability to perform daily chores, 54.7%; capability for work, 64.4%; personal relationships, 57.7%; support from friends, 61%; conditions of the household, 84%; and access to healthcare, 81.4%. Only 36.6% considered their sexual life satisfactory, and about 94% had, at least occasionally, negative feelings. Only 37% reported experiencing enough energy for daily tasks, and 48.6% accepted their physical appearance.

The respondents believed that spirituality comforted and reassured them (73.2%) and found spiritual force in hard times (70.6%), with good connection of body, mind, and spirit (67.8%), even though only 53.4% reported inner peace and 50.7% reported being optimistic. In addition, 72.7% of the respondents reported finding strength in faith, and 44.3% found support in religious or spiritual communities.

Tables 1 and 2 show the beta regression model for the outcomes ‘emotional exhaustion’ and ‘disengagement’, respectively.

**Table 1:** Beta regression model for the dimension of burnout emotional exhaustion, one of the dimensions of Burnout (Oldenburg Burnout Inventory)

Predictive variables	Estimate (95% CI)	p
Workplace (suburb or surroundings of a big city)	0.328 (0.139; 0.516)	0.001 ***
Workplace (mid-sized city)	0.358 (0.105; 0.611)	0.006 **
Workplace (small city)	-0.265 (-0.571; 0.04)	0.089 .
Time allocation for household chores (6-10 hours/week)	-0.05 (-0.184; 0.084)	0.469
Time allocation for household chores (11-20 hours/week)	-0.139 (-0.259; -0.019)	0.023 *
Time allocation for household chores (> 20 hours/week)	-0.122 (-0.226; -0.018)	0.022 *
Wage range (US$ 500-1000)	0.205 (-0.314; 0.724)	0.439
Wage range (US$ 1000-2000)	-0.374 (-0.816; 0.068)	0.097 .
Wage range (US$ 2000-4000)	0.252 (-0.047; 0.552)	0.099 .
Wage range (> US$ 4000)	-0.233 (-0.412; -0.054)	0.011 *
Work environment (poor)	-0.479 (-0.806; -0.153)	0.004 **
Work environment (regular)	0.193 (-0.084; 0.471)	0.172
Work environment (good)	-0.049 (-0.251; 0.152)	0.630
Work environment (excellent)	-0.005 (-0.136; 0.125)	0.937
Quality of life	-0.186 (-0.261; -0.111)	< 0.001 ***
Physical pain	0.06 (-0.004; 0.124)	0.064 .
Need for treatment	0.067 (0.01; 0.124)	0.020 *
Energy	-0.395 (-0.479; -0.311)	< 0.001 ***
Time allocation for leisure	-0.09 (-0.162; -0.019)	0.013 *
Work commute	0.102 (0.038; 0.167)	0.002 **
Capability for work	-0.183 (-0.258; -0.108)	< 0.001 ***
Satisfaction with personal relations	-0.109 (-0.176; -0.042)	0.001 ***
Satisfaction with sexual life	-0.058 (-0.108; -0.008)	0.023 *
Satisfaction with household	0.07 (-0.001; 0.142)	0.055 .
Satisfaction with transportation	-0.156 (-0.233; -0.078)	< 0.001 ***
Negative feelings	0.246 (0.181; 0.311)	< 0.001 ***

Signif. codes: 0 ‘***’ 0.001 ‘**’ 0.01 ‘*’ 0.05 ‘.’ 0.1 ‘ ‘ 1

**Table 2:** Beta regression model for the dimension of burnout disengagement, another dimension of Burnout (Oldenburg Burnout Inventory)

Predictive variables	Estimate (95% CI)	p
Marital status (married or with a partner)	0.231 (0.089; 0.373)	0.001 ***
Marital status (separated or divorced)	0.187 (0.008; 0.366)	0.041 *
Marital status (widow)	0.307 (-0.06; 0.674)	0.101
Workplace (suburb or surroundings of a big city)	0.364 (0.184; 0.544)	< 0.001 ***
Workplace (mid-sized city)	0.239 (-0.008; 0.485)	0.058 .
Workplace (small city)	-0.123 (-0.423; 0.177)	0.422
Workload (21-36 hours)	-0.045 (-0.194; 0.105)	0.560
Workload (37-48 hours)	0.038 (-0.089; 0.166)	0.557
Workload (49-60 hours)	-0.051 (-0.161; 0.059)	0.365
Workload (> 60 hours)	0.084 (-0.008; 0.177)	0.074 .
Time allocation for household chores (6-10 hours/week)	-0.119 (-0.251; 0.013)	0.078 .
Time allocation for household chores (11-20 hours/week)	-0.164 (-0.281; -0.047)	0.006 **
Time allocation for household chores (> 20 hours/week)	-0.238 (-0.338; -0.139)	< 0.001 ***
Time allocation for leisure (6-10 hours/week)	0.167 (-0.032; 0.366)	0.101
Time allocation for leisure (11-20 hours/week)	0.16 (-0.011; 0.33)	0.067 .
Time allocation for leisure (> 20 hours/week)	0.057 (-0.08; 0.194)	0.417
Pandemic income (20% reduction)	-0.141 (-0.282; 0)	0.050 *
Pandemic income (21-50% reduction)	0.016 (-0.119; 0.15)	0.820
Pandemic income (≥ 50% reduction)	0.134 (0.006; 0.262)	0.040 *
Pandemic income (increase)	0.114 (0.005; 0.223)	0.041 *
Work environment (poor)	-0.454 (-0.756; -0.153)	0.003 **
Work environment (regular)	0.01 (-0.242; 0.262)	0.938
Work environment (good)	-0.1 (-0.283; 0.083)	0.283
Work environment (excellent)	0.059 (-0.061; 0.178)	0.336
Physical pain	0.082 (0.027; 0.137)	0.004 **
Concentration	-0.144 (-0.226; -0.062)	0.001 ***
Life safety	-0.142 (-0.225; -0.059)	0.001 ***
Healthy environment	-0.12 (-0.193; -0.047)	0.001 ***
Energy	-0.201 (-0.283; -0.12)	< 0.001 ***
Acceptance of physical appearance	-0.065 (-0.125; -0.005)	0.034 *
Satisfaction with ability to perform daily tasks	0.109 (0.031; 0.187)	0.006 **
Capability for work	-0.249 (-0.332; -0.167)	< 0.001 ***
Satisfaction with sexual life	-0.08 (-0.128; -0.032)	0.001 ***
Caring for others adds meaning to life	-0.236 (-0.311; -0.161)	< 0.001 ***
Optimism	-0.12 (-0.188; -0.052)	0.001 ***

Signif. codes: 0 ‘***’ 0.001 ‘**’ 0.01 ‘*’ 0.05 ‘.’ 0.1 ‘ ‘ 1

For the outcome ‘emotional exhaustion’ ( Table 1 ), the following were significant: place of work; time allocation for household chores; wage range; poor work environment; very poor quality of life; lack of energy for daily tasks; no time allocation for leisure; dissatisfaction with daily work commute. They reported significant dissatisfaction with their: capability for work; transportation; relationship skills; and sexual life. In addition, they reported experiencing negative feelings frequently.

For the outcome ‘disengagement’ ( Table 2 ), the following were significant: marital status; place of work; time allocation for household chores; income reduction/increase during the pandemic; poor work environment; inability to concentrate; feeling unsafe on a daily basis; unhealthy physical environment; lack of energy for daily tasks; non-acceptance of physical appearance; great dissatisfaction with their ability for daily tasks, with their sexual life and with their capability for work; inability to remain optimistic in times of uncertainty; caring for others did not bring meaning to their lives; and considering that physical pain prevented them from doing what needed to be done.

In the classification tree corresponding to the outcome ‘emotional exhaustion’ ( Figure 1 ), the respondents represented in leaves 12 (n=58, 7.5%), 13 (n=43, 5.6%) and 16 (n=35, 4.5%) had the highest scores of burnout, with mean and median equal to or greater than 3.4 (corresponding to 0.8 in the scale from 0 to 1). The respondents represented in leaves 12 and 13 reported experiencing very little or no energy for daily tasks and very frequent negative feelings. These two groups differed regarding their capability for work, and those in leaf 12 reported great dissatisfaction with that. The respondents in leaf 16 reported experiencing little energy for daily tasks and no or very few negative feelings, and not having good quality of life.

**Figure 1:** Classification tree corresponding to the outcome ‘emotional exhaustion,’ one of the dimensions of Burnout (Oldenburg Burnout Inventory). WorkEnvir: work environment; CapWork: capability for work; TreatmNeed: need for medical treatment; QoL: quality of life; NegFeel: negative feelings; PersSatisf: satisfaction with personal relations; SexSatisf: satisfaction with sexual life; TranspSatisf: satisfaction with transportation.

In the classification tree corresponding to the outcome ‘disengagement’ ( Figure 2 ), the respondents represented in leaves 3 (n=97, 12.6%) and 5 (n=29, 3.8%) had the highest scores of burnout, with mean and median over 2.8 (corresponding to 0.6 in the scale from 0 to 1). The 97 respondents in leaf 3 reported dissatisfaction with their capability for work and having very little or no energy for daily tasks. However, the 29 respondents in leaf 5, despite their dissatisfaction with their capability for work and not acknowledging that caring for others brings meaning to life, considered having enough energy for their daily tasks.

**Figure 2:** Classification tree corresponding to the outcome ‘disengagement.’, another dimension of Burnout (Oldenburg Burnout Inventory). CapWork: capability for work; CareOthers: care for others; HealthyEnviron: healthy environment; LifeSafe: life safety; WorkEnvir: work environment.

For the outcome ‘emotional exhaustion’ related to women physicians with burnout, the polychoric correlation coefficient (Supplementary Material 3) identified that having negative feelings had a negative correlation with satisfaction with their sexual life and personal relations, and energy for daily tasks. Requiring medical treatment to cope with daily life correlated negatively with the presence of energy for daily tasks. However, lack of energy for daily tasks correlated positively with poor quality of life and dissatisfaction with capability for work ( Figure 3 ).

**Figure 3:** The polychoric correlation matrix is a network where the nodes were the variables, and the weights on the edges represented the polychoric correlation coefficient. The thickness of the edges and the transparency were given by the correlation coefficient’s magnitude between the nodes. Dimensions of Burnout (Oldenburg Burnout Inventory): (A) Emotional exhaustion without burnout; (B) Emotional exhaustion with burnout; (C) Disengagement without burnout; (D) Disengagement with burnout. (*). With burnout: amS: healthy environment; amT: work environment; cdd: caring for others adds meaning to life; cnc: concentration; cpT: capability for work; enr: energy; otm: optimism in challenging times; qlV: quality of life; sgV: life safety; snN: negative feelings; stP: satisfaction with personal relations; stS: satisfaction with sexual life; stT: satisfaction with transportation; Trt: need for medical treatment. (*) More details for understanding the polychoric correlation matrix can be seen in the supplementary material.

For the outcome ‘disengagement’ related to women physicians with burnout, the polychoric correlation coefficient (Supplementary Material 3) identified that difficulty in concentrating correlated positively with dissatisfaction with capability for work and feeling unsafe on a daily basis. The inability to remain optimistic in times of uncertainty correlated positively with feeling unsafe on a daily basis and the inability to see meaning for their lives in caring for others. The lack of energy for daily tasks correlated positively with dissatisfaction with capability for work ( Figure 3 ). These network analysis findings corroborate those in the classification tree and beta regression.

## Discussion

This study showed a high frequency of burnout among these Brazilian women physicians (61.6%) who answered the survey. Regarding the outcome ‘emotional exhaustion,’ women physicians with burnout have little or no energy for daily tasks, negative feelings, and dissatisfaction with their capability for work. Regarding the outcome ‘disengagement’, women physicians with burnout reported dissatisfaction with their ability for work, little or no energy for daily tasks, and not adding meaning to their lives from caring for others, which threaten their quality of life. Nevertheless, they presented with a relatively good quality of life and believed that spirituality comforted and reassured them in hard times.

Burnout has been defined as a psychological syndrome that results from chronic stress at work, and its key dimensions are exhaustion, cynicism, and lack of professional efficacy.^23^ The condition is compounded by the COVID-19 pandemic that challenges mental health, questions personal beliefs, and threatens the quality of life of HCPs. The problem is aggravated by the household chores traditionally done by women.^24^

Brazil has ranked second in number of COVID-19 cases and deaths. This has stretched the Brazilian healthcare system to the limit, affecting the care provided to patients with not only COVID-19, but other acute and chronic diseases as well. Almost all HCPs, especially physicians, have been involved in the fight against the pandemic.^25^ According to Scheffer et al., Brazil has 477 982 physicians, 222 942 of whom are women, predominantly young, living in the Southeastern, Southern, and Northeastern regions, and most of them (59.5%) having a medical specialist title. Medicine in Brazil is undergoing feminization.^26^ The distribution of our sample’s characteristics is in accordance with that reported in the study by Scheffer et al.

Our study showed a higher prevalence of burnout (61.6%) among Brazilian women physicians as compared to other studies.^1 - 3 , 7 - 9^ The ever-growing number of COVID-19 suspected and confirmed cases, huge workload, shortage of personal protective equipment, overwhelming media coverage, lack of specific medications, and the feeling of insufficient support may be contributing to the psychological damage to HCPs. Many of them feared to get infected or infect their families, friends, and colleagues, and reported high levels of stress, anxiety, and depression symptoms, which may have long-term psychological implications.^27 , 28^

In our study, most respondents reported having good quality of life and believing that their lives had a purpose and that caring for others brought meaning to their lives. In addition, they reported still having to overcome barriers in exercising their profession and receiving lower pay than their male counterparts. A large number reported frequent negative feelings, dissatisfaction with sexual life, and lack of sufficient energy for daily tasks, in addition to dissatisfaction with their physical appearance. Such findings might have been influenced by the effects of the pandemic on a country with a huge challenge to the health system.^25^ Such findings are similar to those of the study with Turkish physicians, reporting that those involved in the fight against COVID-19 reported a strong sense of meaningfulness of work.^29^ However, the frequency of burnout was much higher among Brazilian women physicians, which may be related to the magnitude of the effects of the pandemic in Brazil.

Medical practice is permeated by experiences of loss, stress, anxiety, and fear, which increase the psychological vulnerability of physicians and facilitate the appearance of anxiety-depression symptoms. In addition, resilience, spirituality, and personal beliefs seem to play a mediating role in some of these psychological variables. No study has assessed how these variables affect the exhaustion and psychological suffering of women physicians, mainly those acting in a country facing a broken health system.^30^ We observed that women physicians rely on spirituality for comfort and reassurance, find spiritual strength in challenging times, and believe in getting strength for the daily challenges from faith.

In our study, the novel use of machine learning identified for the outcome ‘emotional exhaustion’ that women physicians with burnout often have little or no energy for daily tasks and negative feelings. For the outcome ‘disengagement’, women physicians reported dissatisfaction with their capability for work, little or no energy for daily tasks, and not adding meaning to their lives from caring for others ( Figures 1 and 2 ). To our knowledge there is no assessment of burnout among women physicians using artificial intelligence techniques, which is the strength of our study.

In addition to confirming the beta regression and classification tree findings of women physicians with burnout, the network analysis ( Figure 3 ) evidenced the correlation of their negative feelings with their dissatisfaction with personal relations and sexual life, as well as with their lack of energy for daily tasks. Moreover, it evidenced the correlation of their poor quality of life with their dissatisfaction with capability for work and their lack of energy for daily tasks, as well as the correlation of their difficulty to concentrate with their feeling of unsafety, which, in turn, correlated with their inability to remain optimistic in times of uncertainty. Moreover, this analysis showed graphically the strength of the relationship between the variables identified in the machine learning technique. Another study using this type of analysis has shown the association of elevated emotional exhaustion with arterial hypertension and other chronic diseases,^6^ even outside the pandemic period.

A limitation of this study is it is a convenience sample, and the high frequency of burnout may be due to a sample bias; women with more problems may have answered the questionnaire more than other women. Although the distribution of the sample’s characteristics is similar to that observed in the study of medical demography in Brazil.^26^ The strength of this study is the joint analysis using machine learning of Burnout conditions, quality of life, and spirituality and their interrelationships during the COVID19 pandemic in female doctors, who faced the most significant challenges in dealing with unique working and living conditions personnel, in a country beset by cases and deaths related to SARS-COV-2 infection.

The pandemic’s socioeconomic effects and how it affects a healthy lifestyle, as well as the female selfcare, well-being sensation, and quality of life represent major threats to the healthcare of women physicians with burnout. Yet, most of the time they are neither acknowledged nor addressed. In countries like Brazil, facing many cases and deaths, this challenge is bigger because of the inequalities of a continental country without target policies for physicians’ health, especially for women physicians challenged by triple workload in pandemic times.

Therefore, it is essential to develop future studies to recognize the prevalence of burnout and its overwhelming impact on different populations to properly address it and prevent it. Our findings highlight the importance of creating an environment conducive to the construction of positive work relationships. In addition, government and healthcare agencies should provide resources and invest to protect healthcare professionals’ psychological well-being by creating mental health programs. In parallel to this, partnerships with other social institutions should be established and remote assistance systems implemented, driven by resilience and comprehension of their unique situation, to help women physicians with burnout.

## Conclusion

This study showed a high frequency of burnout among Brazilian women physicians who answered the survey during the COVID-19 pandemic. Nevertheless, they presented with a relatively good quality of life and believed that spirituality comforted and reassured them in hard times.

## * Supplemental Materials

For additional information Supplemental Material 1, please click here



For additional information Supplemental Material 2, please, click here.



For additional information Supplemental Material 3, please, click here.
